# Evolution of anti-*Trypanosoma cruzi* antibody production in patients with chronic Chagas disease: Correlation between antibody titers and development of cardiac disease severity

**DOI:** 10.1371/journal.pntd.0005796

**Published:** 2017-07-19

**Authors:** Ingebourg Georg, Alejandro Marcel Hasslocher-Moreno, Sergio Salles Xavier, Marcelo Teixeira de Holanda, Eric Henrique Roma, Maria da Gloria Bonecini-Almeida

**Affiliations:** 1 Setor de Imunodiagnóstico, Instituto Nacional de Infectologia Evandro Chagas, Fundação Oswaldo Cruz, Rio de Janeiro, Rio de Janeiro, Brazil; 2 Laboratório de Pesquisa Clínica em Doença de Chagas, Instituto Nacional de Infectologia Evandro Chagas, Fundação Oswaldo Cruz, Rio de Janeiro, Rio de Janeiro, Brazil; 3 Laboratório de Imunologia e Imunogenética em Doenças Infecciosas. Instituto Nacional de Infectologia Evandro Chagas, Rio de Janeiro, Rio de Janeiro, Brazil; US Food and Drug Administration, UNITED STATES

## Abstract

Chagas disease is one of the most important endemic infections in Latin America affecting around 6–7 million people. About 30–50% of patients develop the cardiac form of the disease, which can lead to severe cardiac dysfunction and death. In this scenario, the identification of immunological markers of disease progression would be a valuable tool for early treatment and reduction of death rates. In this observational study, the production of anti-*Trypanosoma cruzi* antibodies through a retrospective longitudinal follow-up in chronic Chagas disease patients´ cohort and its correlation with disease progression and heart commitment was evaluated. Strong inverse correlation (ρ = -0.6375, *p* = 0.0005) between anti-*T*. *cruzi* IgG_1_ titers and left ventricular ejection fraction (LVEF) in chronic Chagas cardiomyopathy (CCC) patients were observed after disease progression. Elevated levels of anti-*T*. *cruzi* IgG_3_ titers were detected in all *T*. *cruzi*-infected patients, indicating a lack of correlation of this IgG isotype with disease progression. Furthermore, low levels of anti-*T*. *cruzi* IgG_2_, IgG_4_, and IgA were detected in all patients through the follow-up. Although without statistical significance anti-*T*. *cruzi* IgE tends to be more reactive in patients with the indeterminate form (IND) of the disease (*p* = 0.0637). As this study was conducted in patients with many years of chronic disease no anti-*T*. *cruzi* IgM was detected. Taken together, these results indicate that the levels of anti-*T*. *cruzi* IgG_1_ could be considered to seek for promising biomarkers to predict the severity of chronic Chagas disease cardiomyopathy.

## Author summary

*Trypanosoma cruzi* is the etiological agent of Chagas disease that affects about 7 million people in Latin America, being considered one of the most important neglected diseases of developing countries. Chronic Chagas disease might be present in different forms as an asymptomatic indeterminate form or even with severe cardiac commitment, known as chronic Chagas cardiomyopathy. In fact, the cardiac form can lead to death due to disease progression. Seeking for biomarkers of cardiomyopathy progression has become important to understand the cardiac progression and to predict or even prevent the disease worsening and to improve the quality of life of affected individuals. In this work, we followed the anti-*T*. *cruzi* antibody profile in a retrospective longitudinal study in a cohort of chronic Chagas disease patients, and further correlate with heart commitment and cardiac disease progression. We found an inverse correlation between anti-*T*. *cruzi* IgG_1_ titers and cardiac disease severity in patients with progressive disease. These data suggest that anti-*T*. *cruzi* IgG_1_ levels could be considered a suitable candidate tool for early identification of cardiac disease progression.

## Introduction

Chagas disease is caused by the flagellate protozoa *Trypanosoma cruzi*, which affects around 6–7 million people in the World, mostly in the Americas [1]. Around 3 million infected people live in Brazil, where the majority of cases are related to the chronic phase of the infection, although new cases of acute infection have been reported in the Brazilian Amazon region [2]. The disease spectrum ranges from a complete absence of symptoms to severe cardiac commitment, depending on the immune response elicited by the host during the disease development [3].

In the chronic phase, indeterminate (IND) patients show an absence of clinical symptoms even presenting positive serology or patent parasitemia for *T*. *cruzi* [4]. This form is responsible for 40% of chronic cases of Chagas disease. About 30–50% of chronic patients develop the cardiac form, presenting from mild to severe cardiac alterations. Patients with chronic Chagas disease cardiomyopathy (CCC) are classified according to the degree of cardiac commitment detected by electro (EKG) or echocardiogram (ECHO) alterations [5].

Studies in past decades attributed to antibodies as a major cause of myocarditis during Chagas disease. According to these studies, molecular mimicry between host and *T*. *cruzi* antigens would elicit the production of antibodies with cross-reactivity, which would be responsible for host tissue damage. Indeed, there are some reports in the literature showing cross-reactivity of serum antibodies from *T*. *cruzi*-infected patients with host proteins [6–8]. However, in recent years, it has been better accepted that the myocardial damage is attributed to an exacerbated host Th1 response, through the production of IFN-γ and TNF-α [9, 10]. The Th1 response might induce an antibody IgG_2_/IgG_3_ isotype switch [11], while a Th2 response promotes mainly the IgG_4_ and IgE isotype switch [12].

Although the exacerbated inflammation is one of the accepted causes for myocarditis, the role of humoral immune response in the progression of Chagas disease is underexplored. The aim of the present work was to identify the kinetics of anti-*T*. *cruzi* antibodies production in a retrospective longitudinal cohort of chronic Chagas patients and correlate with cardiac commitment and disease progression.

## Methods

### Ethics statement

This is a retrospective study and it was not possible to contact all the patients to obtain the informed consent after many years of data collection. However, the study was approved by the Ethical Research Committee of Instituto Oswaldo Cruz /Fiocruz, and the researchers signed to maintain the confidentiality of patients´ medical records.

### Patients, disease stratification, clinical data and sample collection

This is an observational retrospective longitudinal study, where the serology for anti-*T*.*cruzi* IgM, IgG_1-4_, IgE and IgA in patients with chronic Chagas Disease were followed, correlating the levels of antibody titers and cardiac disease severity.

**Fig 1:**
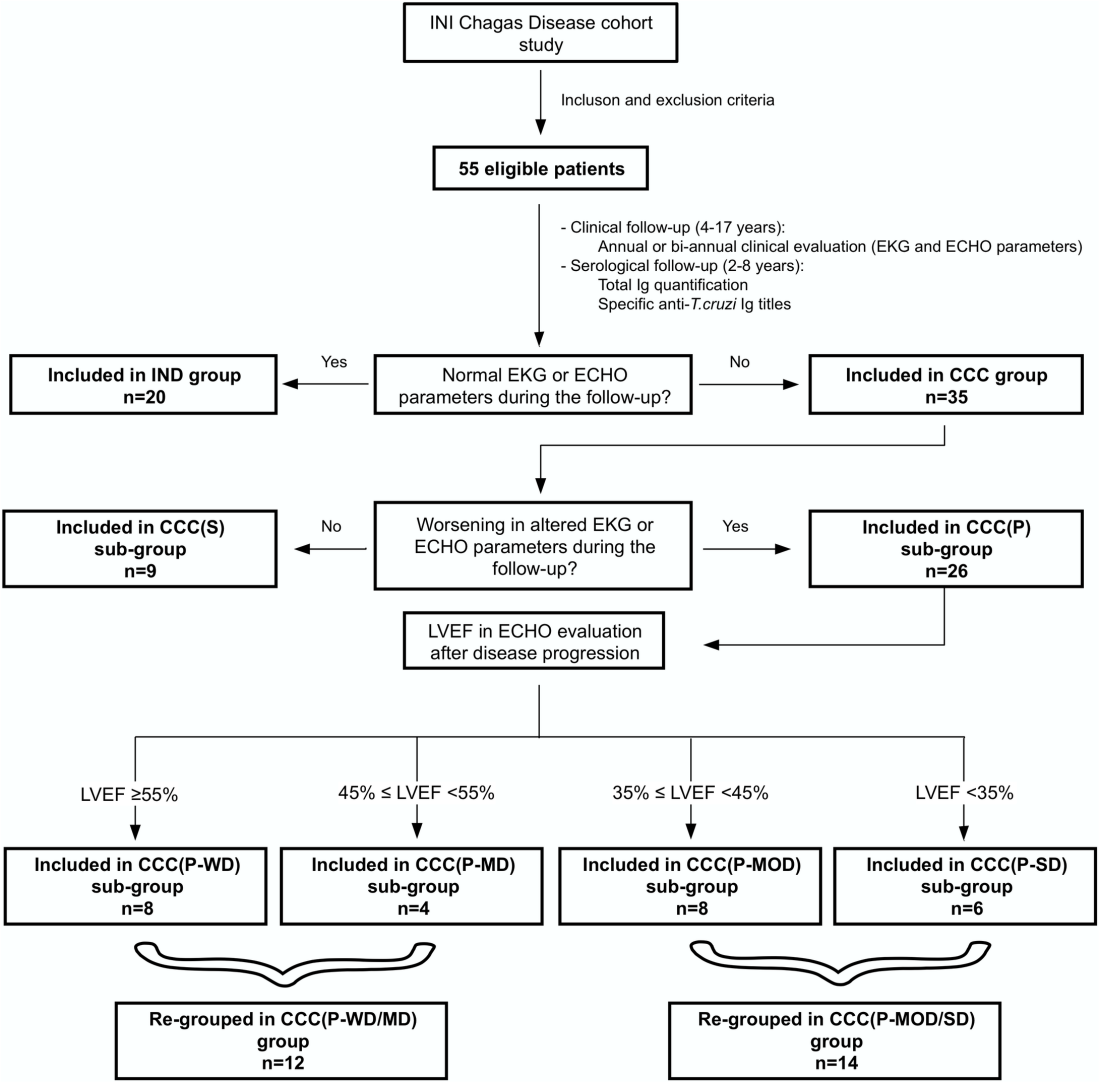
Study flowchart. Strategy used to define groups and sub-groups in the study.

Anti-*T*. *cruzi* antibody levels were determined in sera from 2 to 8 years of serological follow-up from *T*. *cruzi* seropositive chronic Chagas disease patients attended in a clinical cohort at Instituto Nacional de Infectologia Evandro Chagas (INI), Fiocruz, Rio de Janeiro, Brazil. Patients were recruited from 1987 to 2000 and were submitted to an annual or biannual clinical evaluation through 4 to 17 years (1987–2004) of follow-up. During the clinical follow-up, blood samples were obtained for clinical purposes and stored. The number of blood samples collected varied according to each patient follow-up time, from two to eight collections, with a minimum interval of one-year to each other. All samples were kept frozen at -20°C with a maximum of 8 years of storage period. The clinical data was used to classify the patients regarding disease form and cardiac commitment level, at study baseline and during the follow-up, allowing us to identify any cardiac alteration during the study. Therefore, all patients were clinically evaluated since the entry into the study and the last disease classification during the follow-up was used to define the groups and sub-groups. Patients with an indeterminate form of the disease without progression to CCC during the follow-up were included in IND group; patients with stable CCC (without disease progression) were included in the CCC(S) group, and patients with progressive CCC were included in the CCC(P) group. Because of disease progression, CCC(P) patients generated double data, one related to disease stage before progression (BP), and another after progression (AP). Thus, CCC(P) patients were analyzed with data from BP and AP, separately. The disease progression was established as any significant clinical alteration in EKG or ECHO in the patients during the clinical follow-up. The EKG and ECHO were used as disease progression markers, and ECHO was either used to classify disease severity, through left ventricular ejection fraction (LVEF) evaluation. CCC(P) were sub-grouped according to the degree of cardiac commitment severity, based on LVEF values observed after disease progression in: progressive cardiomyopathy without LVEF dysfunction (CCC(P-WD), LVEF ≥55%; Progressive cardiomyopathy with mild LVEF dysfunction (CCC(P-MD), 45≤ LVEF <55%; Progressive cardiomyopathy with moderate LVEF dysfunction (CCC(P-MOD), 35≤ LVEF <45% and Progressive cardiomyopathy with severe LVEF dysfunction (CCC(P-SD), LVEF <35%. Fig 1 shows the study flowchart.

The requirements for study inclusion criteria were: patients of both gender, age higher than 18 years old; showing confirmed diagnosis for Chagas disease by clinical evaluation and two independent serological tests such as ELISA and indirect immunofluorescence assay. Study exclusion criteria were: absence of storage serum that matched the clinical follow-up, age lower than 18 years old and pregnant women during the period of the clinical and serological follow-up.

Clinical data were collected from medical records and include electrocardiogram, echocardiographic, as well as the presence of co-morbidities, such as hypertension, dyslipidemia, obesity, cigar consumption, other cardiovascular diseases and use of medications to control these co-morbidities.

### Electrocardiogram and echocardiographic evaluation

Twelve-lead electrocardiogram was performed for inclusion in the study in allpatients at rest with a long D2 record (30 seconds) for arrhythmias and two observers analyzed tracings independently. In case of disagreement, a consensus diagnosis was obtained after a joint discussion of the electrocardiographic tracing. The analyses were performed blindly, without knowledge of clinical data or other complementary methods. The electrocardiographic changes were classified according to the Minnesota code [13], modified for Chagas disease [14].

Single and two-dimensional echocardiograms with Doppler color were performed in all patients by the same echocardiographer since the week of inclusion in the study. The echocardiography exam included conventional and variations of the conventional cuts, to identify localized segmental changes (usually small mammary aneurysms). The overall left ventricular systolic ejection fraction (LVEF) was estimated objectively by calculations using the Teichholz method [15], being classified as: normal (LVEF ≥55%), slightly depressed (45%≤ LVEF <55%), moderately depressed (35%≤ LVEF <45%) or severely depressed (LVEF <35%) as described before [16].

### Serological samples and determination of total serum immunoglobulins levels

Serial serum samples were conserved at -20°C in the Chagas serum bank in the Laboratory of Immunodiagnostic in INI/Fiocruz until use.

The quantification of total IgG, IgG_1_, IgG_2_, IgG_3_, IgG_4_, IgA, and IgM was performed by automatized Nephelometer ARRAY 360 system (Beckman and Coulter, USA) following manufacturer instructions. The immunoglobulin quantification was expressed as mg/dl, and a standard reference serum was used to calibrate the measurements. The total IgE was quantified by Enzyme-Linked Fluorescent Assay ELFA (Bio-Merieux, Brazil) in the VIDAS (Bio-Merieux) according to manufacturer instructions. The IgE quantification was expressed as international units (IU/L). Highly reactive samples were tested twice for results confirmation.

### Determination of anti-*T*. *cruzi* antibody titers

The anti-*T*. *cruzi* IgG_1_, IgG_2_, IgG_3_ and IgG_4_ isotype titers were measured by Enzyme-Linked Immuno Sorbent Assay (ELISA) kits from Bio-Manguinhos/Fiocruz according to manufacturer instructions with minor modifications. Briefly, 10 μg/ml of proteic soluble antigen from *Y* strain of *T*. *cruzi* epimastigotes were adsorbed in ELISA microplates (Bio-Manguinhos/Fiocruz) for 16 hours at 4°C. The samples were diluted serially starting from 1:100 in phosphate buffer saline 0.05% Tween 20 (PBS-T) plus 5% of fetal bovine serum (FBS) and incubated for 30 minutes at 37°C in an ELISA incubator (Organon Teknika). After extensive wash with PBS-T, monoclonal antibodies anti-human IgG_1_, IgG_2_, IgG_3_ or IgG_4_-horseradish peroxidase (HRP) conjugated, were incubated for 30 minutes at 37°C in the ELISA incubator. Then, the plates were extensively washed, a substrate solution (3,3′,5,5′-Tetramethylbenzidine (TMB), H_2_O_2_ and substrate buffer) was prepared following manufacturer instructions and 100μl were added to wells. The plates were incubated in the dark for 30 minutes at room temperature (RT). The reaction was stopped with H_2_SO_4_ 1N, and the plates were read in a plate reader (PR 2100 Beckman Coulter) at 450 nm.

The detection anti-*T*. *cruzi* IgM, IgE, and IgA were carried with the same procedures as described for IgG isotypes. However, an additional step using the *RF-Absorbent* (Dade Behring), a lyophilized anti-human IgG sheep serum, was performed to block IgG isotypes in the ELISA, following manufacturer instructions. As the concentration of anti-*T*. *cruzi* IgM, IgE and IgA were too low; the results were quantified using reactivity index, which is calculated by the optical density ratio per cut-off value. The cut-off value was determined by the mean of 50 negative sera plus three standard deviations. According to the cut-off value, the samples were considered positive (value above the cut-off) or negative (value below the cut-off). The negative serum was provided by Bio-Manguinhos, Fiocruz.

### Statistical analysis

Differences between patients’ characteristics were calculated by unpaired t-test, Mann-Whitney, ANOVA-one way or Fisher’s exact test. All data were submitted to D’Agostino & Pearson omnibus normality test before statistical calculations. The statistical analysis of anti-*T*. *cruzi* IgG isotypes were calculated using ANOVA-one way plus Kruskal-Wallis post-test for multiple comparisons (IND vs. CCC and sub-groups). The statistical analysis of CCC sub-groups was calculated using ANOVA-two way of repeated measures with Sidak’s multiple comparisons test. Spearman correlation was used to identify association between LVEF and anti-*T*. *cruzi* IgG_1-3_ levels. In parallel, Fisher´s exact test and Bonferroni correction for multiple comparisons were applied to IgE and IgA data to calculate the differences between positivity/negativity between IND and CCC patients. Chi-square test was performed to evaluate the differences in disease classification distribution in CCC(P) patients since the entry into the study. ANOVA-two way of repeated measures with Sidak’s multiple comparisons test was applied to calculate the worsening in the disease classification between CCC(P) patients during the study. All calculations were determined with a *p*-value of <0.05 for significant statistical differences, and the data were plotted as the mean ± standard deviation (SD). The software Graph Pad Prism 7.0a (Graphpad, La Jolla, CA, USA) was used to plot the results and calculate the statistics.

## Results

After clinical analysis at the end of the follow-up, patients remaining as asymptomatic were included in IND group (n = 20); and patients presenting cardiomyopathy were included in CCC group (n = 35). CCC group was sub-grouped according to the presence or absence of disease progression as follows: patients without disease progression were included in CCC(S) sub-group (n = 9); and patients with worsening in cardiac condition, were included in CCC(P) sub-group (n = 26). Moreover, CCC(P) sub-group was stratified according to disease severity based on LVEF measured in ECHO evaluation when disease progression was detected. After this analysis, patients were included in CCC(P) sub-groups as follows: CCC(P-WD), n = 8; CCC(P-MD), n = 4; CCC(P-MOD), n = 8; and CCC(P-SD), n = 6, as demonstrated in Fig 1.

The mean age of patients of CCC group (60.89±12.03 years) was higher when compared to IND patients (49.20±10.54 years), p = 0.0007 (Table 1). No differences were observed about gender, where men composed 40% and 57% of IND and CCC groups, respectively. As expected, IND group presented higher ventricular ejection fraction (70.25±6.70%) compared to CCC group (59.51±9.57%), *p*<0.001. However, both groups presented mean LVEF values above the normal heart function (LVEF≥55%). The serological follow-up was similar between the groups (IND = 4.90±1.48, and CCC = 5.63±1.52 years), with significant difference regarding clinical follow-up (IND = 7.45±3.78, and CCC = 9.97±3.18 years, *p* = 0.0022).

**Table 1:** Chronic Chagas disease patients´ characteristics.

Demographic and clinical characteristics	IND (n = 20)	CCC (n = 35)	*p*-value
Age (years, mean ± SD)	49.20±10.54	60.89±12.03	0.0007^a^
Sex (male, %)	8 (40%)	20 (57%)	0.2695^b^
ECHO (LVEF%) (mean ± SD)	70.25±6.70^a^	59.51±9.57^a^	<0.0001^a^
Serological follow-up (time in years) (mean ± SD)	4.90±1.48	5.63±1.52	0.0899^a^
Clinical follow-up (time in years) (mean ± SD)	7.45±3.78	9.97±3.18	0.0022^a^
Arterial hypertension	2 (10%)	15 (43%)	0.015^b^
Diabetes	0 (0%)	1 (3%)	>0.999^b^
Dyslipidemia	4 (20%)	4 (11%)	0.443^b^
Obesity	0 (0%)	0 (0%)	1.000^b^
Smoker	0 (0%)	1 (3%)	>0.999^b^
Other cardiovascular disease not related to Chagas disease	0 (0%)	0 (0%)	1.000^b^
Total serum Ig levels (mean ± SD)			
IgM (mg/dl)	148.70±58.46	141.20±50.58	0.6193^a^
IgG (mg/dl)	1397.00±310.60	1444.00±254.70	0.5462^a^
IgG_1_ (mg/dl)	826.30±530.30	780.10±267.20	0.6686^a^
IgG_2_ (mg/dl)	365.50±93.33	130.00±21.97	<0.0001^a^
IgG_3_ (mg/dl)	136.10±80.37	373.3±130.00	<0.0001^a^
IgG_4_ (mg/dl)	81.75±57.43	60.36±10.20	0.0354^a^
IgE (IU/L)	525.30±590.7	363.70±833.2	0.4487^a^
IgA (mg/dl)	242.10±120.40	273.40±101.10	0.3077^a^

^a^ *p* values were calculated using unpaired *t*-test

^b^ *p* value was calculated using Fisher´s exact test.

To investigate possible influence in cardiac commitment regardless of Chagas disease, comorbidities associated with cardiomyopathy were analyzed. CCC group presented significant presence of hypertensive patients when compared to IND group, *p* = 0.015 (Table 1). However, other comorbidities such as diabetes, obesity, dyslipidemia, cigar consumption and other non-related Chagas cardiovascular disease were not significant among the groups. Despite the higher number of hypertensive individuals in CCC group, these patients had controlled disease with regular use of specific medications such as anti-diuretics, beta-blockers and angiotensin converting enzyme (S1 Table).

The quantification of total serum immunoglobulins was performed in all patients to analyze possible changes in total antibody production according to disease stage, regardless of the antibody specificity (Table 1). The total levels of IgM, total IgG, IgG_1_, IgA, and IgE were similar between IND and CCC groups, without correlation with total antibody production and development of cardiac disease stage. However, the level of total IgG_2_ and IgG_4_ was higher in IND group (*p*<0.0001 and *p* = 0.0345, respectively), while IgG_3_ was higher in CCC group (*p*<0.0001) (Table 1).

Analysis of CCC patients showed similar characteristics related to age, sex, clinical and serological follow-up and hypertension (Table 2). Before progression (BP), only CCC patients with severe cardiac disease presented differences in LVEF when compared to CCC(S) and CCC(P-WD) sub-groups, while, after disease progression (AP), all sub-groups of CCC(P) were different between them (*p*<0.01). As the LVEF measured by ECHO after disease progression was used to classify CCC(P) sub-groups, these differences were expected. These patients did not present differences related to age, sex, clinical and serological follow-up and hypertension. As only hypertension was statistically different between IND and CCC groups (Table 1), other co-morbidities were not evaluated in these sub-groups.

**Table 2:** Chronic Chagas cardiomyopathy patients´ characteristics.

Characteristics	CCC(S) (n = 9)	CCC(P-WD) (n = 8)	CCC(P-MD) (n = 4)	CCC(P-MOD) (n = 8)	CCC(P-SD) (n = 6)
Age	56.56±13.27	68.25±5.68	65.50±20.42	59.63±12.63	57.50±4.97
Sex, male	6 (67%)	2 (25%)	3 (75%)	4 (50%)	5 (83%)
ECHO (LVEF)–BP	64.67±11.46	67.75±3.24	63.25±10.78	58.38±13.46	43.50±14.43^a^
ECHO (LVEF)–AP^b^	-	66.63±5.29	55.75±5.73	43.25±2.25	28.17±6.27
Serological follow-up (time in years)	5.66±1.94	5.75±1.82	5.25±1.26	5.75±1.83	5.67±1.51
Clinical follow-up (time in years)	9.56±3.28	10.25±3.45	8.00±2.45	11.50±2.14	9.50±4.18
Time to disease progression during the clinical follow-up (years)	-	5.75±2.38	4.00±1.41	7.38±4.00	6.33±2.66
Hypertension	6 (67%)	4 (50%)	2 (50%)	2 (25%)	1 (17%)

^a^ statistical difference compared to CCC(S) and CCC(P-WD) groups (p<0.01)

^b^ all groups were statistically different from each other (p<0.01). The statistical analyses were calculated using ANOVA with Tukey’s multiple comparisons test. The characteristics were represented as mean ± SD, except Sex and Hypertension, where absolute numbers and percentage were used.

To increase the strength of statistical data, CCC patients with progressive disease were re-grouped according to severity of cardiac commitment based on LVEF measurements in CCC(P-WD/MD) (LVEF≥45%, n = 12) and CCC(P-MOD/SD) (LVEF<45%, n = 14). As the sub-groups presented consistent similarities, the analysis of combined groups did not change the data interpretation. The detailed data of these groups are presented in Table 3. Despite the advanced heart damage, CCC(P-MOD/SD) patients are younger than CCC(P-WD/MD) patients (*p* = 0.043) and this last group presented progression defined by EKG changes (*p* = 0.0017), while patients of CCC(P-MOD/SD) showed progression related to ECHO changes (*p* = 0.0002). As expected, because CCC(P-MOD/SD) group presented progression defined by ECHO, these patients showed a more substantial drop in LVEF values when compared to CCC(P-WD/MD), *p* = 0.0024. Despite the type of progression, both groups presented similar time for disease progression. The S2 Table shows the events of EKG or ECHO that characterized progression, the age, the means titers of anti-*T*. *cruzi* IgG_1_ before and after progression, and the time for disease progression for each patient with progressive CCC.

**Table 3:** Characteristics of patients with progressive CCC.

	CCC(P-WD/MD) (n = 12)	CCC(P-MOD/SD) (n = 14)	OR	*p-*value
Age (years)	67.33±11.66	58.14±9.78	-	0.043^a^
Progression defined by EKG	Yes– 11 (91,67%)	Yes– 4 (28,57%)	27.5	0.0017^b^
	No– 1 (8,33%)	No– 10 (71,43%)		
Progression defined by ECHO	Yes– 2 (16,66%)	Yes– 13 (92,86%)	0.015	0.0002^b^
	No– 10 (83,34%)	No– 1 (7,14%)		
LVEF variation (percentage points)	-3.25±7.82	-15.29±12.79	-	0.0024^c^
Time to disease progression (years)	5.17±2.21	6.73±3.37	-	0.16^a^

^a^ *p* values were calculated using unpaired *t*-test

^b^ *p* values were calculated using Fisher’s exact test

^c^ *p* values were calculated using Mann-Whitney test. The values are represented by mean ± standard deviation. The characteristics were represented as mean ± SD for Age, LVEF variation and time to disease progression, while progression defined by EKG or ECHO were represented as absolute numbers and percentage.

As only chronic patients were included in the study, it was expected that all of them would present a negative serology for anti-*T*. *cruzi* IgM. Indeed, none of the patients in this study presented positive serology for anti-*T*. *cruzi* IgM.

**Fig 2:**
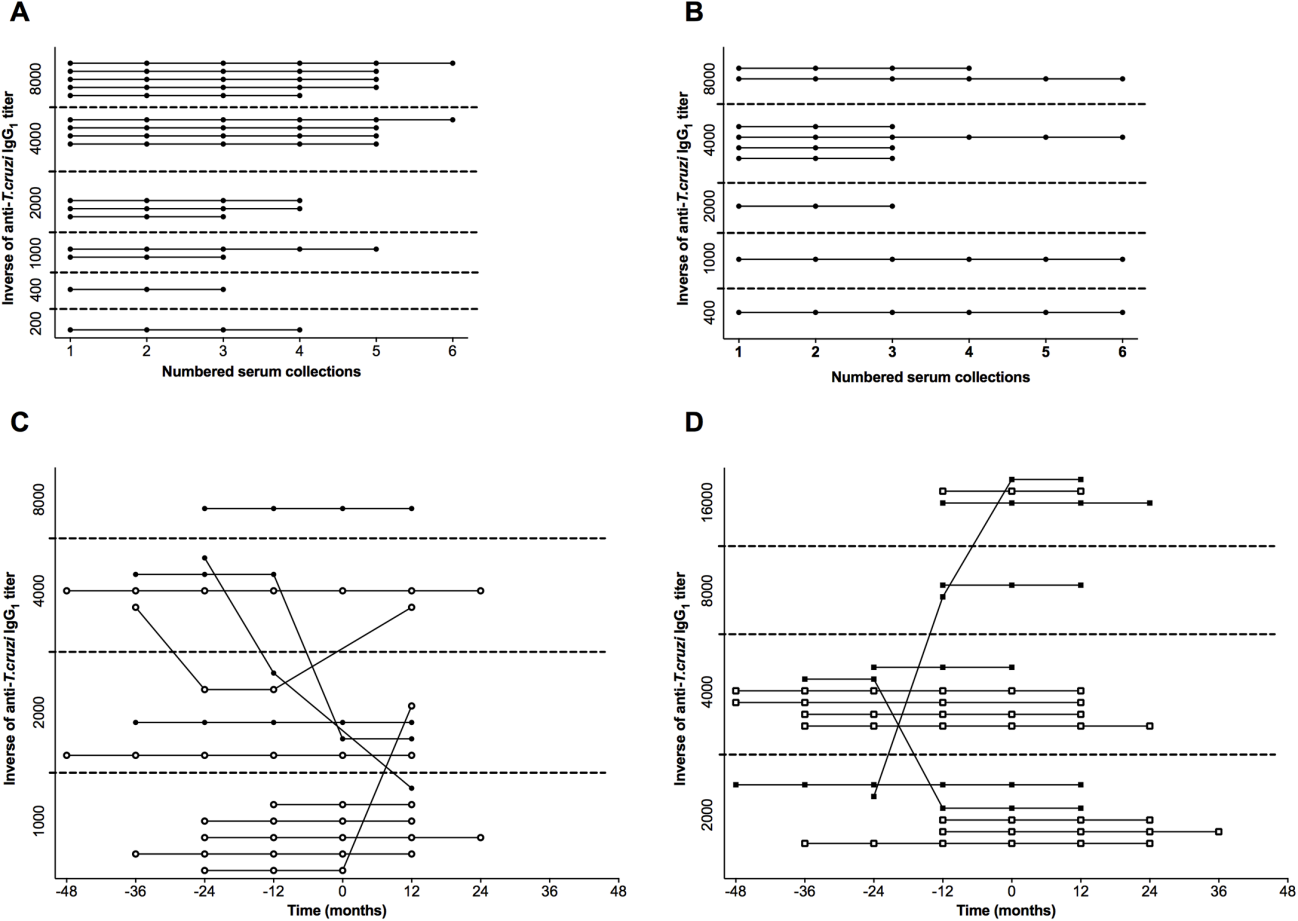
Kinetics of anti-*T*. *cruzi* IgG_1_ during the course of infection in Chagas disease patients. (A) and (B) represent the kinetics of anti-*T*. *cruzi* IgG_1_ titers during the follow-up ordered from first to sixth serum collection for each patient in IND and CCC(S) groups, respectively. Blood samples were obtained sequentially with a minimum of one-year interval between each other. Dashed lines delimitate the range of the antibody titer, represented in the vertical axis. (C) and (D) represent the kinetics of anti-*T*. *cruzi* IgG_1_ titers during the follow-up from 48 months before to 48 months after disease progression for each patient in CCC(P-WD/MD) and CCC(P-MOD/SD) sub-groups, respectively. The time 0 corresponds to the titer measured at the time of disease progression. Open and filled circles represent CCC(P-WD/MD) patients without and with mild LVEF dysfunction, respectively, while open and filled squares represent CCC(P-MOD/SD) patients with moderate and severe LVEF dysfunction, respectively.

**Fig 3:**
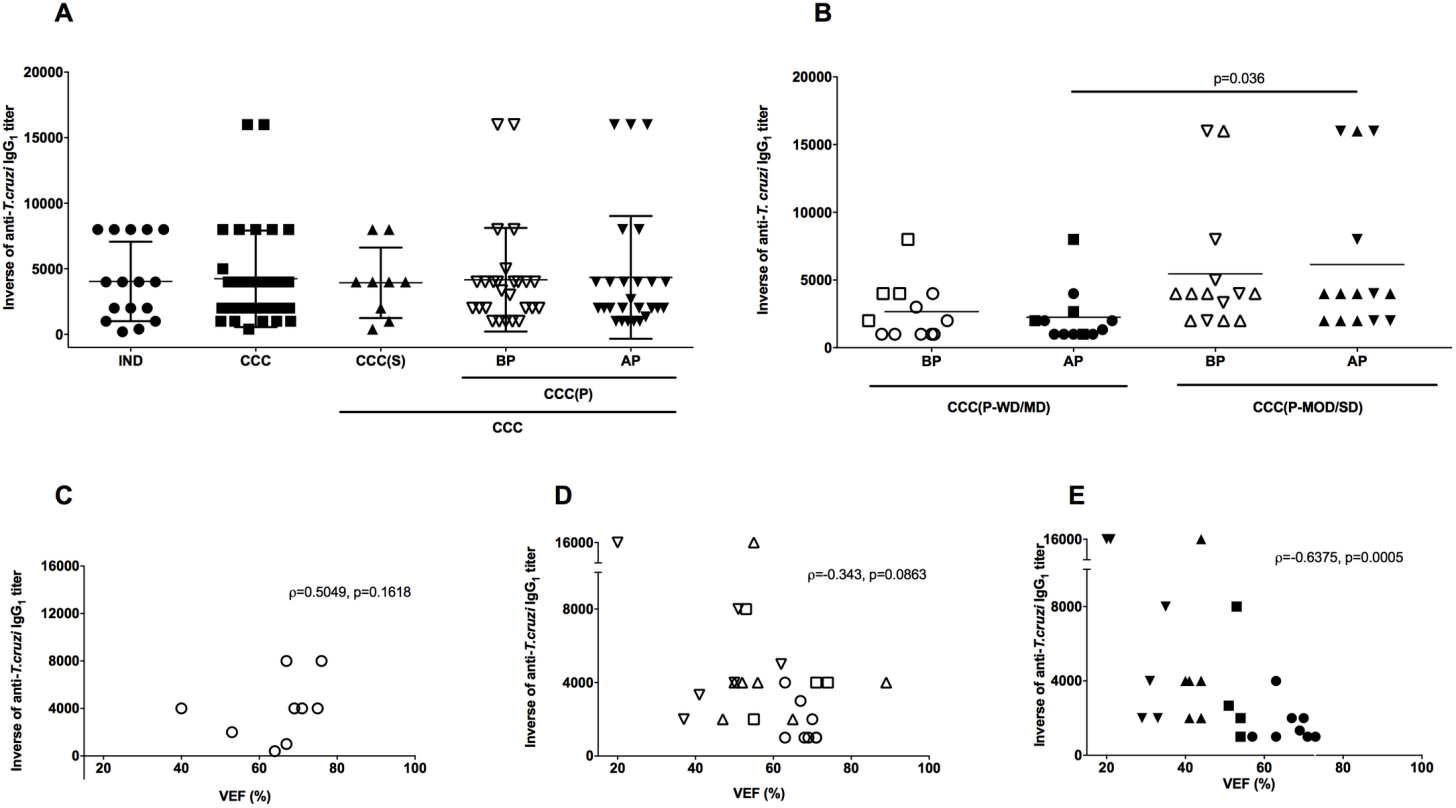
Reactivity of IgG_1_ for anti-*T*. *cruzi* antigen and correlation with ventricular ejection fraction in patients with Chagas disease. (A) Inverse of the anti-*T*. *cruzi* IgG_1_ titers in sera of patients with chronic form of Chagas Disease in the indeterminate form of disease (IND), stable cardiomyopathy (CCC(S)), progressive cardiomyopathy (CCC(P)) before disease progression (BP) and progressive cardiomyopathy after disease progression (AP), respectively. The statistical analysis was calculated using ANOVA-one way plus Kruskal-Wallis post-test for multiple comparisons. (B) Inverse of anti-*T*. *cruzi* IgG_1_ titers in sera of patients with progressive cardiac form of Chagas disease (CCC(P) group) before and after disease progression sub-grouped according to the severity of cardiac commitment. Patients with progressive cardiac disease without (circles) or mild (squares) (CCC(P-WD/MD)) and moderate (triangles) or severe (inverted triangles) (CCC(P-MOD/SD)) cardiac dysfunction before (open symbols) and after (filled symbols) disease progression were represented. The statistical analysis was calculated using ANOVA-two way of repeated measures with Sidak’s multiple comparisons test. The data of A and B were plotted as the mean ± standard deviation (SD). (C, D, and E) Correlation between anti-*T*. *cruzi* IgG_1_ titers and ventricular ejection fraction (LVEF) in patients with the cardiac form of Chagas disease. (C) represents the correlation in patients with stable cardiac disease. (D) represents the correlation in patients with progressive cardiac disease before disease progression. Open circles, squares, triangles, and inverted triangles, represent CCC(P-WD), CCC(P-MD), CCC(P-MOD) and CCC(P-SD) patients, respectively. (E) represents the correlation in patients with progressive cardiac disease after disease progression. Filled circles, squares, triangles, and inverted triangles, represent CCC(P-WD), CCC(P-MD), CCC(P-MOD) and CCC(P-SD) patients, respectively. Spearman correlation was used to identify association between LVEF and anti-*T*. *cruzi* IgG_1_ levels.

To evaluate the kinetics of specific anti-*T*. *cruzi* antibodies during the course of the disease, serological follow-up was performed in parallel with clinical follow-up. The analysis of anti-*T*. *cruzi* IgG_1_ kinetics demonstrated uniformity in the titers of this isotype through serological follow-up in IND and CCC(S) patients (Fig 2A and 2B), despite the large variation in titers between the patients of both groups. In fact, even after six years of serological follow-up, IND and CCC(S) patients kept anti-*T*. *cruzi* IgG_1_ titers constant. In contrast, some patients with progressive cardiomyopathy presented variation in these titers, although not significant considering the groups, during the study (Fig 2C and 2D). Three patients of CCC(P-WD/MD) and two of CCC(P-MOD/SD) groups showed titer variations. The characteristics of these variations were also different among the patients, with some showing increasing, and others decreasing in titers through the study in both groups. Interestingly, the variation in these patients was detected from three years before to one year after disease progression. However, the majority of the CCC(P) patients kept the titers of anti-*T*. *cruzi* IgG_1_ constant before and after disease progression. Similar kinetics patterns were observed to anti-*T*. *cruzi* IgG_2_ and IgG_3_ isotypes (S1 and S2 Figs). Due to the heterogeneity of the individual disease kinetics, each patient with progressive heart disease presented singular characteristics related to time for disease progression. While some patients presented progression in the disease in early years, others presented progression just in the later years of clinical follow-up. Furthermore, due to the retrospective nature of the study using samples provided for clinical purposes, the number of blood collections varied between patients and time. Therefore, this mismatching in disease progression and number of collected samples for each patient generated unbalanced data, which impaired the matched analysis of anti-*T*. *cruzi* IgG_1_ titer kinetics, as shown in Fig 2. Therefore, we calculated the mean value of antibody titers measured during the study for each patient, and we used these data to compare the differences between the groups. For CCC(P) sub-group, two means were calculated, one before (BP) and another after (AP) disease progression (Fig 3). These means were used to analyze the influence of anti-*T*. *cruzi* IgG_1_ titers before and after the disease progression. As the antibody titers did not present significant alterations in patients over time (Fig 2), the normalization of the data did not interfere in the analysis. Similar kinetics were observed in IgG subclasses (IgG_2-3_), where no variation in anti-*T*. *cruzi* titers were observed (S1 and S2 Figs).

The mean titers of anti-*T*. *cruzi* IgG_1_ were similar between CCC and IND patients (Fig 3A). Both groups presented titers around 1:4,000. A detailed analysis of the cardiac group also showed similar titers of anti-*T*. *cruzi* IgG_1_ in CCC(S) and CCC(P) groups, even before or after cardiomyopathy progression (Fig 3A). Indeed, all groups presented similar anti-*T*. *cruzi* IgG_1_ titers. Patients of CCC(P-MOD/SD) sub-group presented higher titers of anti-*T*.*cruzi* IgG_1_ when compared to CCC(P-WD/MD) patients after disease progression (*p* = 0.036) (Fig 3B). However, there is no difference between these groups before disease progression (*p* = 0.17). Statistical differences before (BP) or after (AP) disease progression in both sub-groups of patients with progressive heart disease were not observed. Analysis of CCC patients with stable cardiomyopathy showed no correlation between LVEF and anti-*T*. *cruzi* IgG_1_ titers (ρ = 0.5049, *p* = 0.1618) (Fig 3C). However, the elevated levels of anti-*T*. *cruzi* IgG_1_ seemed to aggravate the cardiac function only in patients with progressive cardiomyopathy. This correlation did not reach statistical significance in these patients before progression (ρ = -0.343, *p* = 0.0863) (Fig 3D). Despite this fact, there is a strong inverse correlation between LVEF and anti-*T*. *cruzi* IgG_1_ titers in CCC(P) sub-group after disease progression (ρ = -0.6375, *p* = 0.0005) (Fig 3E), indicating that cardiac patients with high anti-*T*. *cruzi* IgG_1_ titers tend to have a worsening in the cardiac function during disease progression.

**Fig 4:**
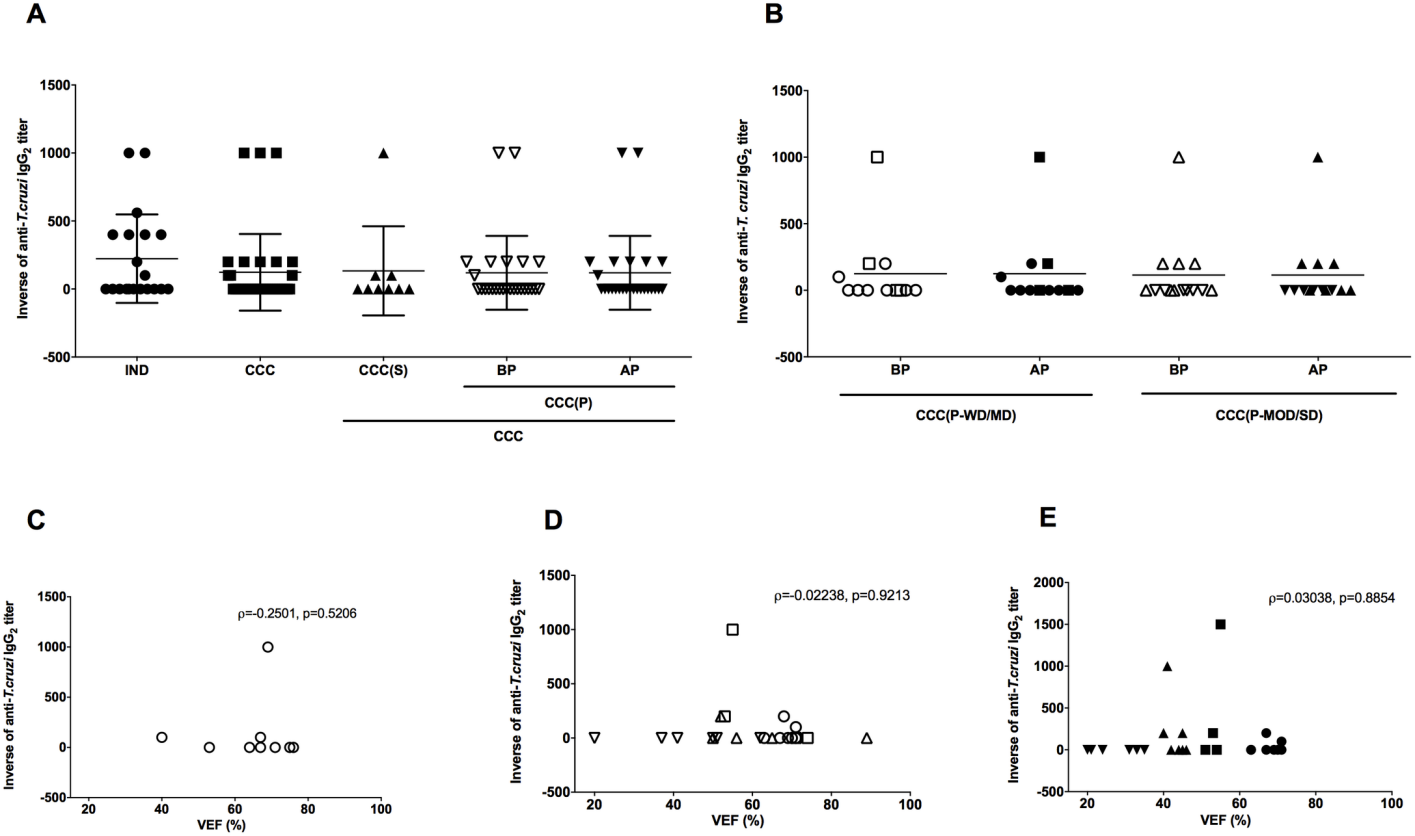
Reactivity of IgG_2_ for anti-*T*. *cruzi* antigen and correlation with ventricular ejection fraction in patients with Chagas disease. (A) Inverse of the anti-*T*. *cruzi* IgG_2_ titers in sera of patients with chronic form of Chagas Disease in the indeterminate form of disease (IND), stable cardiomyopathy (CCC(S)), progressive cardiomyopathy (CCC(P)) before disease progression (BP) and progressive cardiomyopathy after disease progression (AP), respectively. The statistical analysis was calculated using ANOVA-one way plus Kruskal-Wallis post-test for multiple comparisons. (B) Inverse of anti-*T*. *cruzi* IgG_2_ titers in sera of patients with progressive cardiac form of Chagas disease (CCC(P) group) before and after disease progression sub-grouped according to the severity of cardiac commitment. Patients with progressive cardiac disease without (circles) or mild (squares) (CCC(P-WD/MD)) and moderate (triangles) or severe (inverted triangles) (CCC(P-MOD/SD)) cardiac dysfunction before (open symbols) and after (filled symbols) disease progression were represented. The statistical analysis was calculated using ANOVA-two way of repeated measures with Sidak’s multiple comparisons test. The data of A and B were plotted as the mean ± standard deviation (SD). (C, D, and E) Correlation between anti-*T*. *cruzi* IgG_2_ titers and ventricular ejection fraction (LVEF) in patients with the cardiac form of Chagas disease. (C) represents the correlation in patients with stable cardiac disease. (D) represents the correlation in patients with progressive cardiac disease before disease progression. Open circles, squares, triangles, and inverted triangles, represent CCC(P-WD), CCC(P-MD), CCC(P-MOD) and CCC(P-SD) patients, respectively. (E) represents the correlation in patients with progressive cardiac disease after disease progression. Filled circles, squares, triangles, and inverted triangles, represent CCC(P-WD), CCC(P-MD), CCC(P-MOD) and CCC(P-SD) patients, respectively. Spearman correlation was used to identify association between LVEF and anti-*T*. *cruzi* IgG_2_ levels.

The analysis of anti-*T*. *cruzi* IgG_2_ titers showed less reactivity of this isotype when compared to IgG_1_ to *T*. *cruzi* antigens. The highest titer observed of anti-*T*. *cruzi* IgG_2_ was 1:1,000 detected in all groups and sub-groups (Fig 4A) but most of the samples in all groups did not present reactivity for anti-*T*. *cruzi* IgG_2_. Despite the low titers observed in the groups, only CCC(P-SD) patients did not present reactivity for anti-*T*. *cruzi* IgG_2_ (Fig 4B). The analysis between LVEF and anti-*T*. *cruzi* IgG_2_ in cardiac patients did not present any correlation (Fig 4C to 4E), demonstrating a lack of influence of IgG_2_ in the severity of the disease.

**Fig 5:**
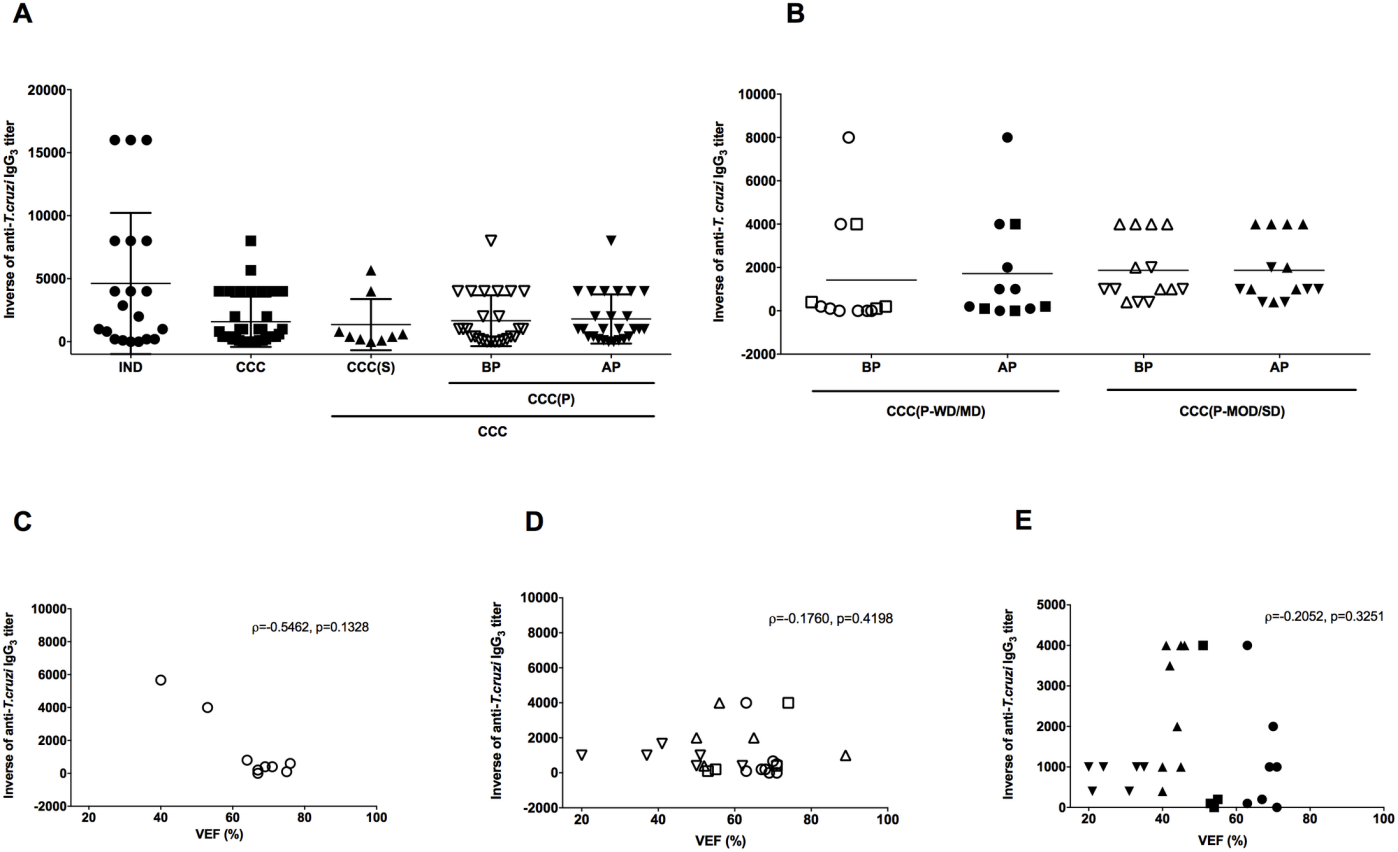
Reactivity of IgG_3_ for anti-*T*. *cruzi* antigen and correlation with ventricular ejection fraction in patients with Chagas disease. (A) Inverse of the anti-*T*. *cruzi* IgG_3_ titers in sera of patients with chronic form of Chagas Disease in the indeterminate form of disease (IND), stable cardiomyopathy (CCC(S)), progressive cardiomyopathy (CCC(P)) before disease progression (BP) and progressive cardiomyopathy after disease progression (AP), respectively. The statistical analysis was calculated using ANOVA-one way plus Kruskal-Wallis post-test for multiple comparisons. (B) Inverse of anti-*T*. *cruzi* IgG_3_ titers in sera of patients with progressive cardiac form of Chagas disease (CCC(P) group) before and after disease progression sub-grouped according to the severity of cardiac commitment. Patients with progressive cardiac disease without (circles) or mild (squares) (CCC(P-WD/MD)) and moderate (triangles) or severe (inverted triangles) (CCC(P-MOD/SD)) cardiac dysfunction before (open symbols) and after (filled symbols) disease progression were represented. The statistical analysis was calculated using ANOVA-two way of repeated measures with Sidak’s multiple comparisons test. The data of A and B were plotted as the mean ± standard deviation (SD). (C, D, and E) Correlation between anti-*T*. *cruzi* IgG_3_ titers and ventricular ejection fraction (LVEF) in patients with the cardiac form of Chagas disease. (C) represents the correlation in patients with stable cardiac disease. (D) represents the correlation in patients with progressive cardiac disease before disease progression. Open circles, squares, triangles, and inverted triangles, represent CCC(P-WD), CCC(P-MD), CCC(P-MOD) and CCC(P-SD) patients, respectively. (E) represents the correlation in patients with progressive cardiac disease after disease progression. Filled circles, squares, triangles, and inverted triangles, represent CCC(P-WD), CCC(P-MD), CCC(P-MOD) and CCC(P-SD) patients, respectively. Spearman correlation was used to identify association between LVEF and anti-*T*. *cruzi* IgG_3_ levels.

High titers of anti-*T*. *cruzi* IgG_3_ were found in patients of all groups, with no statistical difference between groups. IND patients tend to present higher anti-*T*. *cruzi* IgG_3_ titers, when compared to CCC group (*p* = 0.0814) (Fig 5A). Despite this fact, no differences between the cardiac patients with progressive disease in various stages of cardiac commitment were observed (Fig 5B). The lack of association between the severity of the disease and anti-*T*. *cruzi* IgG_3_ was reinforced by the lack of correlation between these antibody titers and LVEF in cardiac patients (Fig 5C to 5E).

**Fig 6:**
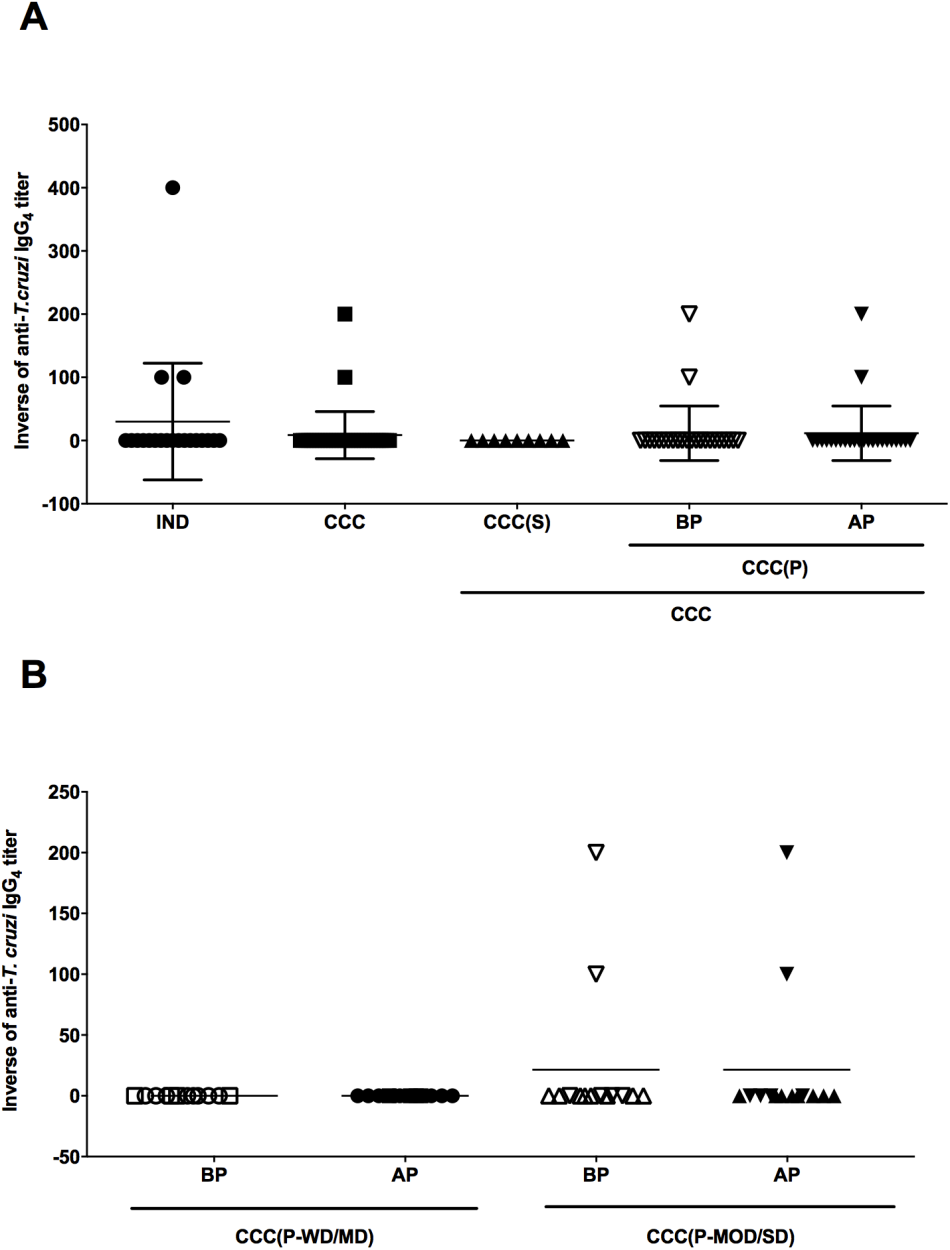
Reactivity of IgG_4_ for anti-*T*. *cruzi* antigen in patients with Chagas disease. (A) Inverse of the anti-*T*. *cruzi* IgG_4_ titers in sera of patients with chronic form of Chagas Disease in the indeterminate form of disease (IND), stable cardiomyopathy (CCC(S)), progressive cardiomyopathy (CCC(P)) before disease progression (BP) and progressive cardiomyopathy after disease progression (AP), respectively. The statistical analysis was calculated using ANOVA-one way plus Kruskal-Wallis post-test for multiple comparisons. (B) Inverse of anti-*T*. *cruzi* IgG_4_ titers in sera of patients with progressive cardiac form of Chagas disease (CCC(P) group) before and after disease progression sub-grouped according to the severity of cardiac commitment. Patients with progressive cardiac disease without (circles) or mild (squares) (CCC(P-WD/MD)) and moderate (triangles) or severe (inverted triangles) (CCC(P-MOD/SD)) cardiac dysfunction before (open symbols) and after (filled symbols) disease progression were represented. The statistical analysis was calculated using ANOVA-two way of repeated measures with Sidak’s multiple comparisons test. The data of A and B were plotted as the mean ± standard deviation (SD).

Anti-*T*. *cruzi* IgG_4_ antibodies were not detectable in most patients, except for three patients in IND group and two patients in the CCC group after disease progression (Fig 6A). Interestingly, these two CCC patients belonged to CCC(P-MOD/SD) sub-group (Fig 6B). Despite the reactivity of five samples, the maximum titer of anti-*T*. *cruzi* IgG_4_ observed was 1:200 and these titers were constant during the study, indicating that this antibody isotype does not seem to be important during the disease progression. Consequently, there is no correlation between of anti-*T*. *cruzi* IgG_4_ titers and LVEF.

The majority of IND (65%) patients presented positivity for specific anti-*T*. *cruzi* IgE against only 9% of CCC patients (*p* = 0.0637) (Table 3). This difference was significant in CCC(S) group when compared with IND group (*p* = 0.0084). Interestingly, none of the patients in CCC(S) group presented positivity for anti-*T*. *cruzi* IgE. No statistical difference between CCC(P) group (before and after disease progression) and CCC(S) group was observed, as well as between CCC(P) patients before and after disease progression. (Table 4).

**Table 4:** IgE reactivity to *T*. *cruzi* antigen.

IgE reactivity / groups	Positive	Negative	Total	OR	*p*-value	Corrected *p*-value*
IND	13 (65%)	7 (35%)	20	5.365^a^	0.0091^a^	0.0637^a^
				34.20^b^	0.0012^b^	0.0084^b^
				3.508^c^	0.0731^c^	0.5117^c^
				2.971^d^	0.1362^d^	0.9534^d^
CCC	9 (26%)	26 (74%)	35	-	-	-
CCC(S)	0 (0%)	9 (100%)	9	0.09695^c^	0.0742^c^	0.5194^c^
				0.08271^d^	0.0363^d^	0.2541^d^
CCC(P)—BP	9 (35%)	17 (65%)	26	0.8471^d^	1.0000^d^	NS^d^
CCC(P)—AP	10 (38%)	16 (62%)	26	-	-	-

^a^ compared to CCC group

^b^ compared to CCC(S) group

^c^ compared to CCC(P)–BP

^d^ compared to CCC(P)–AP. The statistical differences were calculated by Fisher´s exact test.

* Bonferroni correction for multiple comparisons. NS = not significant.

Anti-*T*. *cruzi*-specific IgA antibody reactivity was similar between all groups analyzed, and most patients were negative (Table 5). As IgA antibody is correlated with the digestive form of Chagas disease, and the patients included in this study did not present this form of the disease, we expected low reactivity of this isotype in the study.

**Table 5:** IgA reactivity to *T*. *cruzi* antigen.

IgA reactivity / groups	Positive	Negative	Total	OR	*p*-value	Corrected *p*-value*
IND	6 (30%)	14 (70%)	20	0.8214^a^	0.7755^a^	NS^a^
				1.5000^b^	1.0000^b^	NS^b^
				0.6857^c^	0.7557^c^	NS^c^
				0.9643^d^	1.0000^d^	NS^d^
CCC	12 (34%)	23 (66%)	35	-	-	-
CCC(S)	2 (22%)	7 (88%)	9	0.4571^c^	0.4496^c^	NS^c^
				0.6429^d^	1.0000^d^	NS^d^
CCC(P)—BP	10 (39%)	16 (61%)	26	1.4060^d^	0.7712^d^	NS^d^
CCC(P)—AP	8 (31%)	18 (69%)	26	-	-	-

^a^ compared to CCC group

^b^ compared to CCC(S) group

^c^ compared to CCC(P)–BP

^d^ compared to CCC(P)–AP. The statistical differences were calculated by Fisher´s exact test.

* Bonferroni correction for multiple comparisons. NS = not significant.

**Fig 7:**
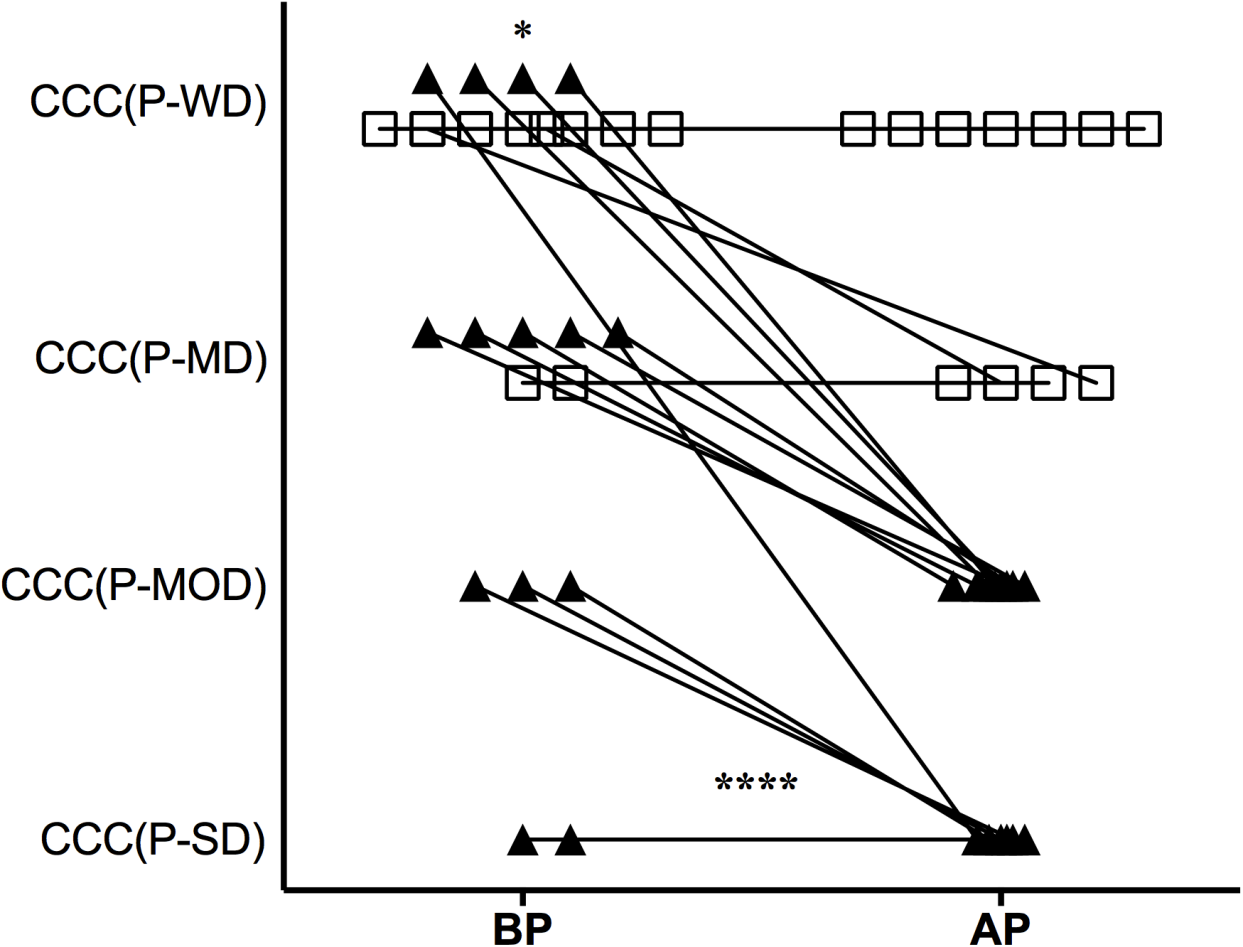
Disease classification in CCC(P) patients before and after disease progression. Disease classification from entry into the study (BP) to after disease progression (AP) in CCC(P) sub-groups are represented (horizontal axis). The vertical axis represents the levels of disease severity according to LVEF values as described in Methods. Connecting lines link the patients’ disease classification BP and AP disease progression. Open squares represent CCC(P-WD/MD) patients, while filled triangles represent CCC(P-MOD/SD) patients. *Statistical difference between CCC(P-WD/MD) and CCC(MOD/SD) sub-groups BP calculated by chi-square test, *p* = 0.033). **** Statistical difference between CCC(P-WD/MD) and CCC(MOD/SD) sub-groups calculated by paired ANOVA-two way with Sidak’s multiple comparisons test, *p*<0.0001. To perform this calculation the vertical axes were transformed in arbitrary units where: CCC(P-WD) = 4; CCC(P-MD) = 3; CCC(P-MOD) = 2; and CCC(P-SD) = 1.

We further evaluated how cardiac disease progressed in CCC(P-WD/MD) and CCC(P-MOD/SD) sub-groups during the disease outcome, taking the disease classification at the entry into and at the end of the study (Fig 7). Patients of CCC(P-WD/MD) sub-group did not show a worsening in disease severity, whereas they kept LVEF constant during the follow-up (*p* = 0.63), opposite to observed to CCC(P-MOD/SD) patients, which showed significant worsening in disease severity (*p*<0.0001). In fact, only two patients of CCC(P-WD/MD) sub-group showed a decrease in LVEF sufficient to change them in disease classification until the end of the study. Both progressed from absence to mild LVEF dysfunction. In contrast, twelve patients of CCC(P-MOD/SD) sub-group showed a worsening in disease classification during the clinical follow-up. This change in disease classification was even worse in CCC(P-MOD/SD) where these patients presented a fall greater than one level in disease classification. Furthermore, the two patients without disease worsening in CCC(P-MOD/SD) sub-group already presented a worse disease condition even in the earlier cardiac evaluation. Analysis of CCC(P-WD/MD) and CCC(P-MOD/SD) groups showed differences in disease classification distribution since the entry into the study. CCC(P-MOD-SD) patients showed more diversity in disease classification, while CCC(P-WD/MD) were restricted to mild stages of the cardiac disease (p = 0.03). S2 Table shows detailed information related to LVEF measurements in patients before and after disease progression.

## Discussion

Humoral immune response and clinical outcome of Chagas disease patients attended at INI/Fiocruz with 4 to 17 years of clinical follow-up and 2 to 8 years of annual serological data collection were followed. Around 50% of CCC patients presented disease progression with cardiac alteration at EKG or ECHO levels during the clinical follow-up. The cut-off values of LVEF for determining CCC(P) sub-groups were based on the American Heart Association guidelines, adapted to Chagas disease as previously described [16]. This study was conducted in 2004, therefore before the implementation of the first Brazilian Chagas Consensus (BCC) in 2005, which is the guideline for Chagas disease classification [17]. For this reason, the current disease classification was not applied in this study. Regardless of some differences in methods, the disease severity classification used in this study and BCC share similarities. Applying current disease classification, CCC(P-WD/MD) patients would be classified as stage A and B1, while CCC(P-MOD/SD) patients would be classified as stage B2, C, and D of BCC classification. In fact, the cut-off values of LVEF ≥45% and LVEF <45% are used to classify CCC patients in A/B1 and B2/C/D disease stage in BBC, respectively, and the same values were used to group CCC(P-WD/MD) and CCC(P-MOD/SD) patients in this study.

IND patients were younger than CCC patients, and could generate a bias in the study. To match the period in which IND and CCC patients were clinically followed, we looked forward in clinical records for cardiac progression in IND group after five years of segment, and none of them had changed their clinical cardiac status, maintaining as IND form of chronic Chagas disease.

Reports in the literature addressing the role of specific anti-*T*. *cruzi* antibody production in Chagas disease and correlation with severity of the cardiac form of the disease are scarce. Studies in experimental model showed production of specific antibodies from acute to chronic phase of the *T*. *cruzi*- infection [18, 19]. The resistant B10 mouse strain produces higher levels of anti-*T*. *cruzi* IgM compared to the susceptible C3H background in the acute phase of infection [20]. Anti-*T*. *cruzi* IgM specific antibodies were absent in all patients in this study. This specific antibody isotype occurs primarily in the acute phase of the infection, with a switch for IgG isotypes during the chronic phase of the infection [21–23]. However, data in the literature have shown production of this antibody isotype even in the chronic phase of the infection in human disease [24, 25]. These contradictory findings could be explained based on geographic distribution of patients, re-infections, or even differences in *T*. *cruzi* strain. The cited studies either included patients living in endemic areas, where cases of re-infection could occur increasing anti-*T*. *cruzi* IgM levels in serum. The present cohort study follows patients that had left the endemic area for more than 5 years, which considerably decreases the possibility of re-infection. Another explanation could be associated with the differences in the method for antibody detection, such as specific *T*. *cruzi* antigens used as target for IgM detection assay.

*T*. *cruzi*-specific IgG isotypes have been described as important mechanisms to control parasitemia by the formation of IgG-parasite microaggregates, complement opsonization and platelet activation, which would facilitate the internalization of the parasites by phagocytic cells [26]. In mice experimentally infected model, IgG_1_ and IgG_2_ isotypes have an important role in parasite killing, despite IgG_2_ presents higher specificity to the antigen when compared to IgG_1_ [27]. IgG has been described as a marker of Chagas disease progression. Zauza and Borges-Pereira [28] showed increasing levels of anti-*T*. *cruzi* IgG antibodies in CCC patients over 10 years of clinical follow-up, and this isotype might influence clinical outcome. Unfortunately, the authors did not analyze specific IgG isotypes, impairing the analysis of the relationship between clinical progression and a specific IgG subtype. In our study, no differences in total IgG_1_ production were observed, demonstrating similar patterns of total IgG_1_ production among patient groups. Therefore, any possible difference in anti-*T*. *cruzi* igG_1_ titers could be attributed to the specific immune response elicited to parasites without interference of host IgG_1_ overall production. The kinetics of anti-*T*. *cruzi* IgG_1_ titers were kept mostly constant during the entire study. As mentioned before our cohort participants immigrated to metropolitan Rio de Janeiro, where a low probability of re-infection is expected, as well as no significant changes in anti-*T*. *cruzi* specific antibodies induced by antigen immune re-stimulation. In fact, only six of fifty-five patients presented changes in anti-*T*. *cruzi* IgG_1_ titers during the study. Interestingly, these six patients belonged to CCC(P) group, which indicates additional factors contributing to specific anti-*T*. *cruzi* antibody level changes in some individuals with CCC. An association was observed between the levels of specific anti-*T*. *cruzi* IgG_1_ and LVEF, indicating that elevated levels of specific IgG_1_ could be involved in the cardiomyopathy progression. Interestingly, this association seems to be restricted to patients with progressive cardiac disease, since the same correlation in patients with CCC(S) was not observed. Possible additional factors, such as cytokines, could act in consonance with specific anti-*T*. *cruzi* IgG_1_ antibody production promoting disease progression in CCC patients. To our knowledge, there are no reports describing the role and relationship of cytokines and anti-*T*. *cruzi* IgG_1_ in Chagas patients. However, Pissetti, et al. [29] showed that elevated levels of IL-10 correlated with elevated levels of total IgG_4_ in the serum of patients with the digestive form of Chagas disease, indicating that IL-10 could contribute to total IgG_4_ production in these patients. In fact, low production of anti-*T*. *cruzi* IgG_4_ in IND or CCC patients was detected.

In our study, no substantial changes in anti-*T*. *cruzi* IgG_1_ titers during the follow-up in all groups were observed. Interestingly, the patients with more severe progressive cardiac disease presented higher titers of anti-*T*. *cruzi* IgG_1_. Therefore, the initial elevated levels of anti-*T*. *cruzi* IgG_1_ could predict a worse prognosis in disease progression in patients with progressive cardiomyopathy. In contrast, Cordeiro et al. [30] showed that anti-*T*.*cruzi* IgG_1_ present more reactivity in IND compared to CCC patients. This discordance might be explained because of the use of different antigen sources in the studies. In this study, soluble epimastigote antigens were used as source of *T*. *cruzi* antigens, while Cordeiro et al. used fixed trypomastigote forms of *T*. *cruzi* parasites. Parasites in different life cycle forms express diverse antigens and can elicit different immune responses for each specific form.

In order of antibody isotype reactivity, it was found that IgG_1_ is the predominant antibody produced against *T*. *cruzi* antigens, followed by IgG_3_, IgG_2_, and IgG_4_, respectively. These results agreed with the data observed in the literature about anti-*T*. *cruzi* IgG isotype production, despite some little differences related to order of quantified production of IgG_2_ and IgG_3_ [30–32]. In contrast to results observed of specific anti-*T*. *cruzi* IgG isotypes, the total quantification of IgG_2_ and IgG_3_ presented many differences between groups, where IgG_2_ and IgG_3_ were higher in IND and CCC, respectively. As differences in specific anti-*T*. *cruzi* IgG_2_ or IgG_3_ were not observed, there is no association of these total IgG isotypes with disease form. Many factors might induce specific IgG isotype production, such as other comorbidities not evaluated in this study. Therefore, additional data are needed to associate levels of specific IgG isotypes with Chagas disease development.

The majority of IND patients presented sustained production of anti-*T*. *cruzi* IgE antibodies and only a few CCC patients presented IgE reactivity to *T*. *cruzi* antigens in this study. Although the Bonferroni correction changed the statistical significance between IND and CCC groups, it may point to further studies in large case-control studies to clarify the role of anti-*T*. *cruzi* IgE and their regulators in Chagas disease. Data about anti-*T*. *cruzi* IgE antibody production in patients with Chagas disease and its correlation with disease progression are very rare in the literature. Mineo et al. [33] described a significant correlation between specific anti-*T*. *cruzi* IgE in pericardium fluid of patients with CCC, being present only in the samples from patients with the cardiac form of Chagas disease. Unfortunately, the authors did not report the peripheral level of this isotype in their volunteers. Anti-leishmanial IgE antibodies were described as a marker of active disease in visceral leishmaniasis [34]. The production of IgE specific antibodies is associated with the development of a skewed Th2 response, induced by IL-4 production and downregulated in the presence of IFN-γ [35, 36]. In fact, the imbalance Th1 response, promote heart damage in CCC patients [9, 37]. Therefore, our data suggested that an anti-*T*. *cruzi* IgE in IND patients may downregulate the development of a strong Th1 response, and inhibit heart tissue damage. Further analysis may address this association in clinical sets to confirm the possible use of anti-*T*. *cruzi* IgE as a marker for disease progression.

Our data suggest a lack of correlation between IgA production and severity or progression of cardiac disease. Few patients, independent of clinical stage showed positivity to anti-*T*. *cruzi* IgA antibody. In fact, the IgA levels seem to be relevant in patients with chronic digestive form of the disease [38, 39]. Studies addressing the levels of anti-*T*.*cruzi* IgA in patients with digestive form could reveal the possible use of this isotype as a biomarker for the digestive form of the disease, since this isotype was barely detected in this study.

Analysis of the type of disease progression in CCC(P) patients showed differences in the events that triggered this progression. While CCC(P-WD/MD) showed predominance of EKG alterations, CCC(P-MOD/SD) showed more frequent ECHO alterations. Also, the type of progression (detected by EKG or ECHO) did not seem to interfere in the time to disease progression, since CCC(P-WD/MD) and CCC(P-WD/MD) had similar time to present cardiac alterations. The antibody titers did not change considerably during the study, showing that the immune response elicited before was responsible for disease progression in these patients. Therefore, in this study, patients with higher titers of anti-*T*. *cruzi* IgG_1_, even many years before the decline in cardiac function, tend to have disease progression through a decrease in cardiac function as LVEF worsens. More studies addressing the role of anti-*T*. *cruzi* IgG_1_ antibodies in myocardial fiber damage might elucidate the results here presented.

Many associated comorbidities might affect heart function. In this study, we considered the influence of arterial hypertension, obesity, diabetes, dyslipidemias, cigar consumption and other cardiovascular disease not related to Chagas disease. Only arterial hypertension showed a possible influence in CCC patients because 43% of these patients were hypertensive, against only 10% of IND patients. However, all patients had controlled arterial hypertension due to regular use of specific medications. In fact, the regular use of these medications could even delay disease progression as they are also used for heart protection, such as the beta-blockers. In addition, no differences between CCC sub-groups were observed related to arterial hypertension, indicating that this specific comorbidity did not affect CCC progression. Possibly, other factors not evaluated here contribute to decreasing levels of LVEF in CCC(P-MOD/SD) patients, such as genetic background (including of the parasite), immune response, stress, nutrition, among others, regardless of the significant involvement of anti-*T*.*cruzi* IgG_1_ in this process. Therefore, more cohort studies are needed to identify co-morbidities that might contribute to LVEF decreasing in Chagas disease.

Despite promising results about the possible use of anti-*T*. *cruzi* specific antibodies as markers of CCC progression, we would indicate some limitations in the present work. As this work was conducted as a retrospective study using already collected clinical data and blood samples for clinical purposes, the follow-up period in IND and CCC groups was not possible to be matched. Due to the extended time involved in the clinical follow-up, it would be hard to track many patients for an extended period. Moreover, there are few Chagas cohort studies that are capable of following a large number of patients matched by age, sex, period of clinical and serological follow-up to complete the gaps identified in our study. Certainly, additional studies considering these biases could confirm the data here presented.

This study contributes to a better understanding of how the specific anti-*T*. *cruzi* antibodies are produced in the course of Chagas disease infection in humans. In addition, it opens the possibility of considering anti-*T*. *cruzi* antibody titers as possible targets in studies seeking for biomarkers of cardiac disease severity and progression. In this context, the knowledge about antibody production and its influence during the disease progression might clarify better strategies for prognostic and earlier treatments for cardiomyopathy in Chagas disease.

## Supporting information

S1 Checklist STROBE checklist. (DOC)

S1 Table Patients using medications to control cardiovascular diseases. (DOC)

S2 Table Characteristics of CCC patients with progressive disease. (DOC)

S1 Fig Kinetics of anti-*T*. *cruzi* IgG_2_ in course of infection in Chagas disease patients. (A) and (B) represent the kinetics of anti-*T*. *cruzi* IgG_2_ titers during the follow-up ordered from first to sixth serum collection for each patient in IND and CCC(S) groups, respectively. Blood samples were obtained sequentially with a minimum of one-year interval between each other. Dashed lines delimitate the range of the antibody titer, represented in the vertical axis. (C) and (D) represent the kinetics of anti-*T*. *cruzi* IgG_1_ titers during the follow-up from 48 months before to 48 months after disease progression for each patient in CCC(P-WD/MD) and CCC(P-MOD/SD) sub-groups, respectively. The time 0 corresponds to the titer measured at the time of disease progression. Open and filled circles represent CCC(P-WD/MD) patients without and with mild LVEF dysfunction, respectively, while open and filled squares represent CCC(P-MOD/SD) patients with moderate and severe LVEF dysfunction, respectively.
(TIFF)

S2 Fig Kinetics of anti-*T*. *cruzi* IgG_3_ in course of infection in Chagas disease patients. (A) and (B) represent the kinetics of anti-*T*. *cruzi* IgG_3_ titers during the follow-up ordered from first to sixth serum collection for each patient in IND and CCC(S) groups, respectively. Blood samples were obtained sequentially with a minimum of one-year interval between each other. Dashed lines delimitate the range of the antibody titer, represented in the vertical axis. (C) and (D) represent the kinetics of anti-*T*. *cruzi* IgG_1_ titers during the follow-up from 48 months before to 48 months after disease progression for each patient in CCC(P-WD/MD) and CCC(P-MOD/SD) sub-groups, respectively. The time 0 corresponds to the titer measured at the time of disease progression. Open and filled circles represent CCC(P-WD/MD) patients without and with mild LVEF dysfunction, respectively, while open and filled squares represent CCC(P-MOD/SD) patients with moderate and severe LVEF dysfunction, respectively.
(TIF)
