# Supplementary material for: Evolution of anti-Trypanosoma cruzi antibody production in patients with chronic Chagas disease: Correlation between antibody titers and development of cardiac disease severity
Source: PLoS Negl Trop Dis. 2017 Jul 19;11(7):e0005796. doi: 10.1371/journal.pntd.0005796 (PMC5536389; doi:10.1371/journal.pntd.0005796)
Supplement: S1 Checklist — (DOC) [file pntd.0005796.s001.doc]

STROBE Statement—Checklist of items that should be included in reports of ***cohort studies***

**Evolution of anti-*Trypanosoma cruzi* antibody production in patients with Chronic Chagas Disease: correlation between antibody titers and development of cardiac disease severity**

|  | Item No | Recommendation | Comments |
| --- | --- | --- | --- |
| **Title and abstract** | 1 | (*a*) Indicate the study’s design with a commonly used term in the title or the abstract | See abstract |
|  |  | (*b*) Provide in the abstract an informative and balanced summary of what was done and what was found | See abstract |
| Introduction | | |  |
| Background/rationale | 2 | Explain the scientific background and rationale for the investigation being reported | See Introduction |
| Objectives | 3 | State specific objectives, including any prespecified hypotheses | See Introduction (last paragraph) and Methods sections (Paragraph 1) |
| Methods | | |  |
| Study design | 4 | Present key elements of study design early in the paper | See Methods section (Patients, disease stratification, clinical data and sample collection) |
| Setting | 5 | Describe the setting, locations, and relevant dates, including periods of recruitment, exposure, follow-up, and data collection | See Methods section (Patients, disease stratification, clinical data and sample collection) |
| Participants | 6 | (*a*) Give the eligibility criteria, and the sources and methods of selection of participants. Describe methods of follow-up | See Methods section (Patients, disease stratification, clinical data and sample collection) |
|  |  | (*b*) For matched studies, give matching criteria and number of exposed and unexposed | Not applicable |
| Variables | 7 | Clearly define all outcomes, exposures, predictors, potential confounders, and effect modifiers. Give diagnostic criteria, if applicable | See Methods section (Patients, disease stratification, clinical data and sample collection) |
| Data sources/ measurement | 8* | For each variable of interest, give sources of data and details of methods of assessment (measurement). Describe comparability of assessment methods if there is more than one group | See Methods section |
| Bias | 9 | Describe any efforts to address potential sources of bias | See Results section (Paragraph 8) |
| Study size | 10 | Explain how the study size was arrived at | See Methods section (Patients, disease stratification, clinical data and sample collection) |
| Quantitative variables | 11 | Explain how quantitative variables were handled in the analyses. If applicable, describe which groupings were chosen and why | See Methods section (Electrocardiogram and Echocardiographic evaluation; Serological samples and determination of total serum immunoglobulins levels; Determination of anti-T. cruzi antibodies titles) |
| Statistical methods | 12 | (*a*) Describe all statistical methods, including those used to control for confounding | See Methods section (Statistical analysis) |
|  |  | (*b*) Describe any methods used to examine subgroups and interactions | See Methods section (Statistical analysis) |
|  |  | (*c*) Explain how missing data were addressed | See Results section (Paragraph 8) |
|  |  | (*d*) If applicable, explain how loss to follow-up was addressed | There was no loss to follow-up |
|  |  | (*e*) Describe any sensitivity analyses | See Methods section (Statistical analysis) |
| Results | | |  |
| Participants | 13* | (a) Report numbers of individuals at each stage of study—eg numbers potentially eligible, examined for eligibility, confirmed eligible, included in the study, completing follow-up, and analysed | See results section (Paragraph 1) |
|  |  | (b) Give reasons for non-participation at each stage | Not applicable |
|  |  | (c) Consider use of a flow diagram | See Fig 1 |
| Descriptive data | 14* | (a) Give characteristics of study participants (eg demographic, clinical, social) and information on exposures and potential confounders | See results section (Paragraph 2, 3, and 5) and Tables 1 and 2 |
|  |  | (b) Indicate number of participants with missing data for each variable of interest | See results section (Fig 2, S1 and S2) |
|  |  | (c) Summarise follow-up time (eg, average and total amount) | See results section (Table 1) |
| Outcome data | 15* | Report numbers of outcome events or summary measures over time | See results section (Paragraph 1) and Fig 7 |
| Main results | 16 | (*a*) Give unadjusted estimates and, if applicable, confounder-adjusted estimates and their precision (eg, 95% confidence interval). Make clear which confounders were adjusted for and why they were included | See results section (Tables 1-5; Figures 1-6; Supporting information Tables S1and S2, and Figures S1 and S2) |
|  |  | (*b*) Report category boundaries when continuous variables were categorized | Not applicable |
|  |  | (*c*) If relevant, consider translating estimates of relative risk into absolute risk for a meaningful time period | Not applicable |
| Other analyses | 17 | Report other analyses done—eg analyses of subgroups and interactions, and sensitivity analyses | See results section (Fig 7) |
| Discussion | | |  |
| Key results | 18 | Summarise key results with reference to study objectives | See discussion section (Paragraph 1-3; 5-10) |
| Limitations | 19 | Discuss limitations of the study, taking into account sources of potential bias or imprecision. Discuss both direction and magnitude of any potential bias | See discussion section (Paragraph 2 and 12) |
| Interpretation | 20 | Give a cautious overall interpretation of results considering objectives, limitations, multiplicity of analyses, results from similar studies, and other relevant evidence | See discussion section (Paragraph 12) |
| Generalisability | 21 | Discuss the generalisability (external validity) of the study results | See discussion section (Paragraph 12 and 13) |
| Other information | | |  |
| Funding | 22 | Give the source of funding and the role of the funders for the present study and, if applicable, for the original study on which the present article is based | This study was financially supported by Fiocruz.  The funders had no role in any part of this work from study design to preparation of the manuscript |

*Give information separately for exposed and unexposed groups.

**Note:** An Explanation and Elaboration article discusses each checklist item and gives methodological background and published examples of transparent reporting. The STROBE checklist is best used in conjunction with this article (freely available on the Web sites of PLoS Medicine at http://www.plosmedicine.org/, Annals of Internal Medicine at http://www.annals.org/, and Epidemiology at http://www.epidem.com/). Information on the STROBE Initiative is available at http://www.strobe-statement.org.
