# Supplementary material for: Evolution of anti-Trypanosoma cruzi antibody production in patients with chronic Chagas disease: Correlation between antibody titers and development of cardiac disease severity
Source: PLoS Negl Trop Dis. 2017 Jul 19;11(7):e0005796. doi: 10.1371/journal.pntd.0005796 (PMC5536389; doi:10.1371/journal.pntd.0005796)
Supplement: S1 Table — (DOC) [file pntd.0005796.s002.doc]

**S1 Table. Patients using medications to control cardiovascular diseases**

| **Medications** | **IND (n=20)** | **CCC (n=35)** |
| --- | --- | --- |
| ACE inhibitors | 1 (5%) | 7 (20%) |
| β-blockers | 2 (10%) | 2 (5.7%) |
| Diuretics | 0 (0%) | 1 (2.86%) |
| Vasodilators | 0 (0%) | 1 (2.86%) |
| ACE inhibitors  β-blockers  Diuretics | 0 (0%) | 6 (17.1%) |
| ACE inhibitors  β-blockers | 0 (0%) | 3 (8.6%) |
| ACE inhibitors  Diuretics | 0 (0%) | 3 (8.6%) |
| ACE inhibitor  Vasodilators | 0 (0%) | 2 (5.7%) |

ACE = Angiotensin converting enzyme. The data represent the absolute numbers of patients using specific medications.
