# Supplementary material for: Evolution of anti-Trypanosoma cruzi antibody production in patients with chronic Chagas disease: Correlation between antibody titers and development of cardiac disease severity
Source: PLoS Negl Trop Dis. 2017 Jul 19;11(7):e0005796. doi: 10.1371/journal.pntd.0005796 (PMC5536389; doi:10.1371/journal.pntd.0005796)
Supplement: S2 Table — (DOC) [file pntd.0005796.s003.doc]

**S2 Table. Characteristics of CCC patients with progressive disease**

| **Patient/Group** | **Age (years)** | **EKG (BDP)** | **EKG (ADP)** | **ECHO (BDP)** | **ECHO (ADP)** | **Progression defined by** | **Time to disease progression during the study**  **(in years)** | **IgG_1_ titer (Inverse of mean-BDP)** | **IgG_1_ titer (Inverse of mean-ADP)** |
| --- | --- | --- | --- | --- | --- | --- | --- | --- | --- |
| CCC(P-WD)  1 | 70 | RBBB2  LAH | RBBB2  LAH  **AVB2**  **PI** | AA with VEF still normal  VEF 63% | VEF 63% | EKG | 3 | 4000 | 4000 |
| CCC(P-WD)  2 | 70 | RBBB3 LAH | RBBB3  LAH  **BSA**  **VPRC** | VEF 69% | VEF 69% | EKG | 6 | 1000 | 1333 |
| CCC(P-WD)  3 | 66 | RBBB2 | RBBB2 I**VES**  **AVB1** | VEF 71% | VEF 57% | EKG | 8 | 1000 | 1000 |
| CCC(P-WD)  4 | 60 | RBBB2 | RBBB2  **LAH** | VEF 71% | VEF 71% | EKG | 2 | 1000 | 1000 |
| CCC(P-WD)  5 | 74 | RBBB2 LAH | RBBB2  LAH  **AVB2**  **PI** | VEF 67% | VEF 67% | EKG | 9 | 3000 | 2000 |
| CCC(P-WD)  6 | 73 | RBBB2 LAH | RBBB2  LAH  **VPRC** | VEF 68% | VEF 73% | EKG | 7 | 1000 | 1000 |
| CCC(P-WD)  7 | 73 | IVES | IVES  **RBBB3**  **LAH** | VEF 63% | VEF 63% | EKG | 6 | 1000 | 1000 |
| CCC(P-WD)  8 | 60 | RBBB2 | RBBB2  **LAH** | VEF 70% | VEF 70% | EKG | 5 | 2000 | 2000 |
| CCC(P-MD)  1 | 49 | RBBB2 LAH | RBBB2  LAH | VEF 74% | **VEF 51%** | ECHO | 3 | 4000 | 2666 |
| **Patient/Group** | **Age (years)** | **EKG (BDP)** | **EKG (ADP)** | **ECHO (BDP)** | **ECHO (ADP)** | **Progression defined by** | **Time to disease progression during the study**  **(in years)** | **IgG_1_ titer (Inverse of mean-BDP)** | **IgG_1_ titer (Inverse of mean-ADP)** |
| CCC(P-MD)  2 | 69 | NORMAL | **Multifocal FVES** | VEF 53% | VEF 53% | EKG | 6 | 8000 | 8000 |
| CCC(P-MD)  3 | 51 | RBBB1 AVB1 | AVB1G+ RBBB 1 G+ **FVES** | VEF 55% | 55% | EKG | 3 | 2000 | 2000 |
| CCC(P-MD)  4 | 93 | LAH | LAH  **LBBB2**  **VPRC** | VEF 71% | **VEF 54%** | ECHO + EKG | 4 | 4000 | 1000 |
| CCC(P-MOD)  1 | 59 | RBBB3  LAH | RBBB3  LAH  **AVB1**  **PI** | VEF 65% | **VEF 44%** | ECHO + EKG | 4 | 2000 | 2000 |
| CCC(P-MOD)  2 | 36 | RBBB2 LAH | RBBB2  LAH | VEF 50% | **VEF 41%** | ECHO | 5 | 4000 | 4000 |
| CCC(P-MOD)  3 | 74 | PR | PR  **IVES** | VEF 47% | **VEF 41%** | ECHO + EKG | 8 | 2000 | 2000 |
| CCC(P-MOD)  4 | 50 | RBBB2 LAH | RBBB2  LAH | VEF 52% | **VEF 40%** | ECHO | 13 | 4000 | 4000 |
| **Patient/Group** | **Age (years)** | **EKG (BDP)** | **EKG (ADP)** | **ECHO (BDP)** | **ECHO (ADP)** | **Progression defined by** | **Time to disease progression during the study**  **(in years)** | **IgG_1_ titer (Inverse of mean-BDP)** | **IgG_1_ titer (Inverse of mean-ADP)** |
| CCC(P-MOD)  5 | 59 | RBBB3  LAH  FVES  VPRC | RBBB3  LAH  FVES  VPRC | VEF 53% | **VEF 44%** | ECHO | 5 | 2000 | 2000 |
| CCC(P-MOD)  6 | 72 | RBBB2  LAH  AVBT  PI | RBBB2  LAH  AVBT  PI | VEF 56% | **VEF 45%** | ECHO | 10 | 4000 | 4000 |
| CCC(P-MOD)  7 | 67 | LBBB3 AVB1 | LBBB3  AVB1 | VEF 55% | **VEF 45%** | ECHO | 2 | 16000 | 16000 |
| CCC(P-MOD)  8 | 52 | RBBB1 | RBBB1 | VEF 89% | **VEF 45%** | ECHO | 12 | 4000 | 4000 |
| CCC(P-SD)  1 | 59 | RBBB2  LAH | RBBB2  LAH | VEF 41% | **VEF 29%** | ECHO | 5 | 3333 | 2000 |
| CCC(P-SD)  2 | 63 | RBBB1 | RBBB1 **LBBB2**  **LAH**  **IEZ** | VEF 20% | VEF 20% | EKG | 5 | 16000 | 16000 |
| **Patient/Group** | **Age (years)** | **EKG (BDP)** | **EKG (ADP)** | **ECHO (BDP)** | **ECHO (ADP)** | **Progression defined by** | **Time to disease progression during the study**  **(in years)** | **IgG_1_ titer (Inverse of mean-BDP)** | **IgG_1_ titer (Inverse of mean-ADP)** |
| CCC(P-SD)*  3 | 61 | RBBB3 | RBBB3  **LAH**  **VPRC** | NORMAL VEF 62% | **VEF 50% VEF 21%** | ECHO +EKG | 8 | 5000 | 16000 |
| CCC(P-SD)  4 | 59 | RBBB2  LAH  VPRC | RBBB2  LAH  VPRC | VEF 50% | **VEF 31%** | ECHO | 4 | 4000 | 4000 |
| CCC(P-SD)*  5 | 50 | PR | PR | VEF 51% | **VEF 45% VEF 35%** | ECHO | 11 | 8000 | 8000 |
| CCC(P-SD)  6 | 53 | AVB1  LBBB2  IVES | AVB1  LBBB2  IVES | VEF 37% | **VEF 33%** | ECHO | 5 | 2000 | 2000 |

AA = Apical akinesia; ADP = After disease progression; AVB = atrioventricular block (grade 1 or 2 or total); BDP = Before disease progression; BSA = bradycardia with sinus arrest; FVES = Frequent ventricular extrasystole; IEZ = inactive electrical zone; IVES = Isolated ventricular extrasystole; LAH = Left anterior hemiblock; LBBB = left bundle branch block (grade 1, 2 or 3); PI = pacemaker implantation; PR = pacemaker rhythm; RBBB = right bundle branch block (grade 1, 2 or 3); TGAVB = third-degree atrioventricular block; VPRC = Ventricular primary repolarization changes; * Patients presenting two events of disease progression.
