# Supplementary figures and images for: Evolution of anti-Trypanosoma cruzi antibody production in patients with chronic Chagas disease: Correlation between antibody titers and development of cardiac disease severity
Source: PLoS Negl Trop Dis. 2017 Jul 19;11(7):e0005796. doi: 10.1371/journal.pntd.0005796 (PMC5536389; doi:10.1371/journal.pntd.0005796)

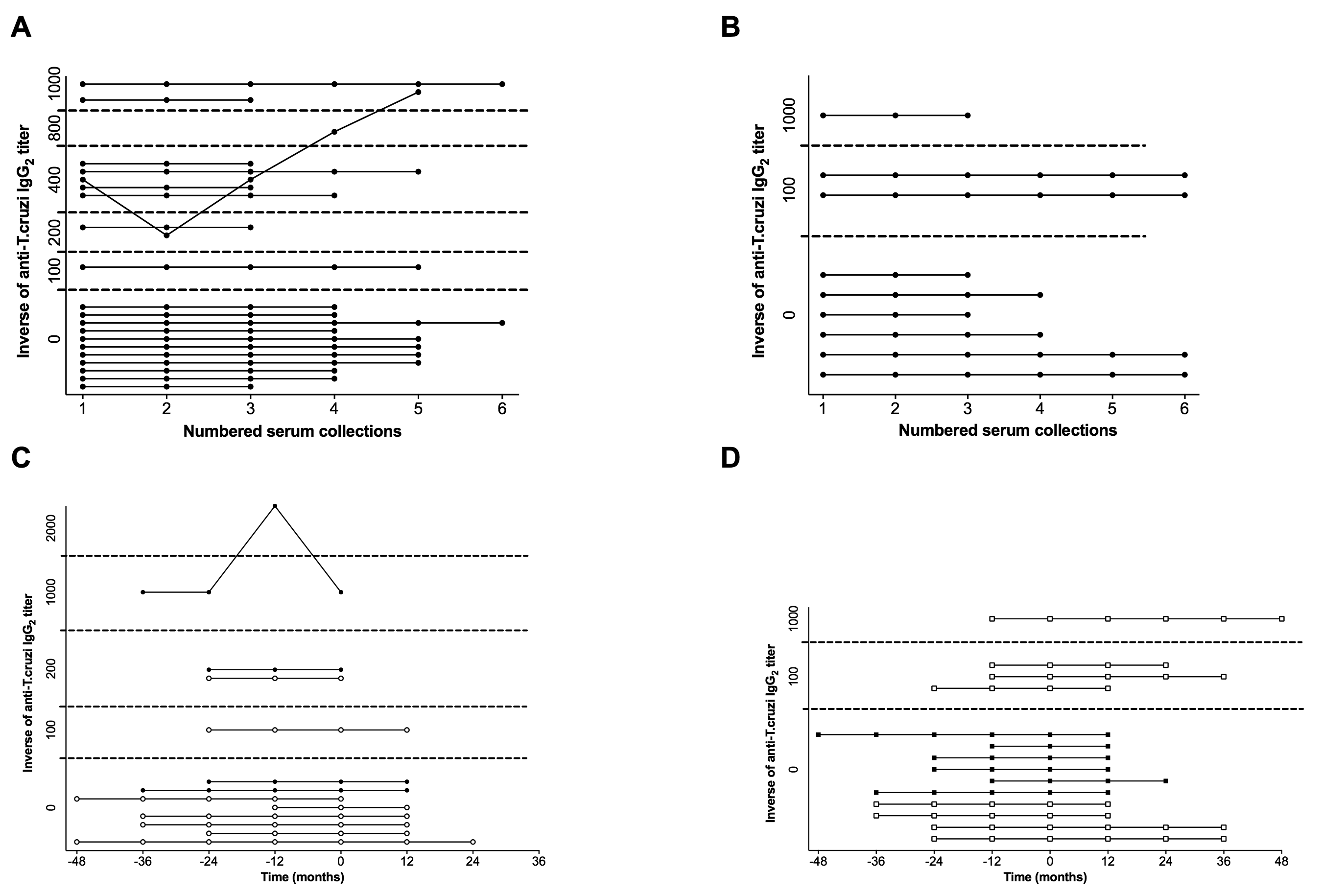

Supplement: S1 Fig — (A) and (B) represent the kinetics of anti-T. cruzi IgG2 titers during the follow-up ordered from first to sixth serum collection for each patient in IND and CCC(S) groups, respectively. Blood samples were obtained sequentially with a minimum of one-year interval between each other. Dashed lines delimitate the range of the antibody titer, represented in the vertical axis. (C) and (D) represent the kinetics of anti-T. cruzi IgG1 titers during the follow-up from 48 months before to 48 months after disease progression for each patient in CCC(P-WD/MD) and CCC(P-MOD/SD) sub-groups, respectively. The time 0 corresponds to the titer measured at the time of disease progression. Open and filled circles represent CCC(P-WD/MD) patients without and with mild LVEF dysfunction, respectively, while open and filled squares represent CCC(P-MOD/SD) patients with moderate and severe LVEF dysfunction, respectively. (TIFF) [file pntd.0005796.s004.tiff]

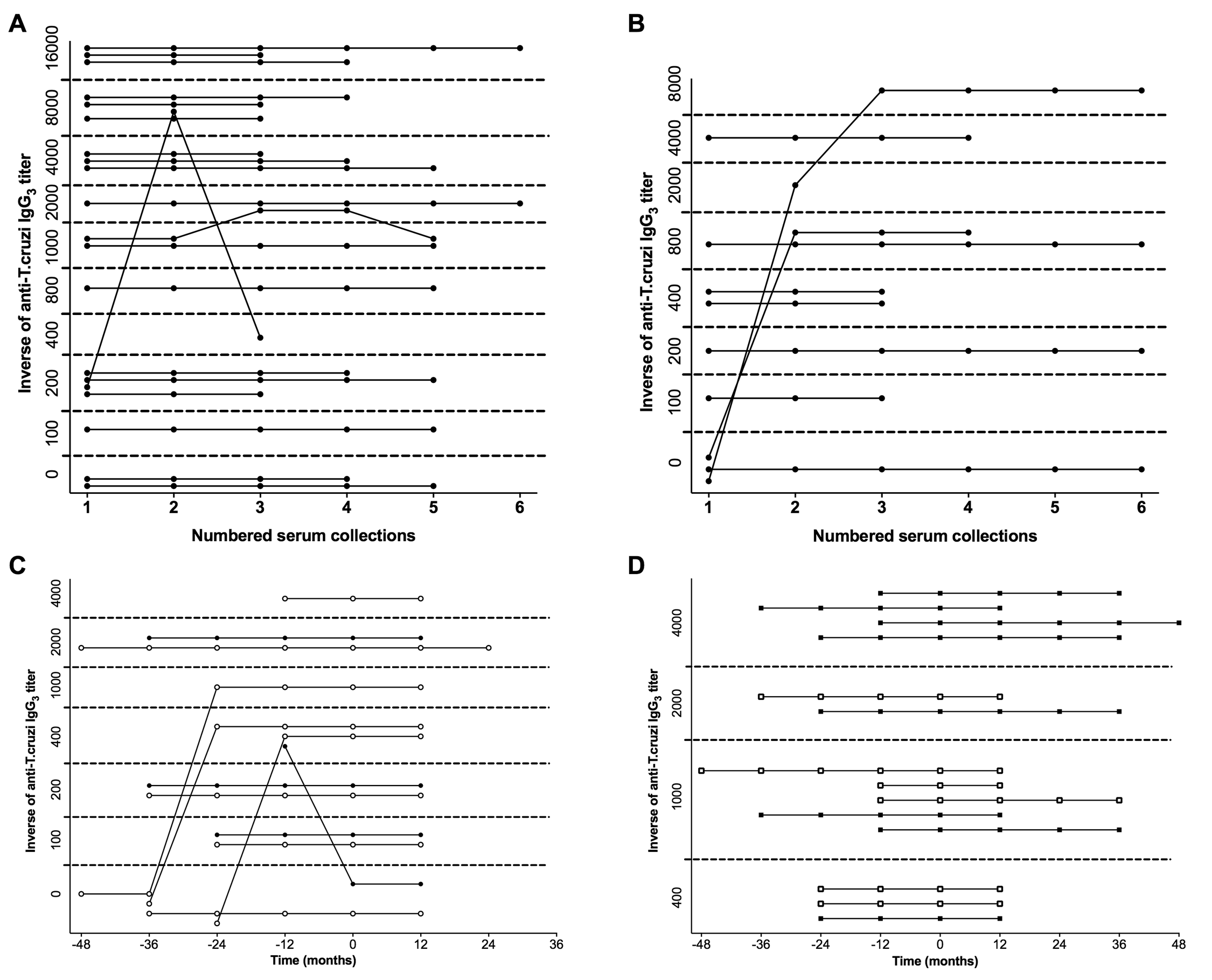

Supplement: S2 Fig — (A) and (B) represent the kinetics of anti-T. cruzi IgG3 titers during the follow-up ordered from first to sixth serum collection for each patient in IND and CCC(S) groups, respectively. Blood samples were obtained sequentially with a minimum of one-year interval between each other. Dashed lines delimitate the range of the antibody titer, represented in the vertical axis. (C) and (D) represent the kinetics of anti-T. cruzi IgG1 titers during the follow-up from 48 months before to 48 months after disease progression for each patient in CCC(P-WD/MD) and CCC(P-MOD/SD) sub-groups, respectively. The time 0 corresponds to the titer measured at the time of disease progression. Open and filled circles represent CCC(P-WD/MD) patients without and with mild LVEF dysfunction, respectively, while open and filled squares represent CCC(P-MOD/SD) patients with moderate and severe LVEF dysfunction, respectively. (TIF) [file pntd.0005796.s005.tif]
